# A Complex Network-Based Approach for Detecting and Characterizing Power Neurons in Drosophila

**DOI:** 10.1007/s12021-026-09773-6

**Published:** 2026-02-13

**Authors:** Enrico Corradini, Federica Parlapiano, Giorgio Terracina, Domenico Ursino

**Affiliations:** 1 DII, Polytechnic University of Marche, Ancona, Italy; 2 DEMACS, University of Calabria, Rende (CS), Italy

**Keywords:** Drosophila melanogaster, Connectome, Power neurons, Connectome motifs, Complex network analysis, Neuron backbone

## Abstract

Connectome analysis investigates the connections in the brain to understand how brain regions communicate with each other and how brain structure relates to its function. In recent years, researchers have reconstructed the structural connectome of several organisms, the most complex being *Drosophila melanogaster*. Two research groups have reconstructed the larval and adult connectomes of this organism and have applied network analysis to learn more about the *Drosophila* brain and its behavior. In this paper, we aim to continue the work of these two research groups at the larval and adult stages. Specifically, we construct several derived network representations and define a set of techniques that use the main concepts and measures of complex network analysis to extract new knowledge about *Drosophila* connectomes at the larval and adult stages. First, we conduct an Exploratory Data Analysis on the larval and adult connectomes to detect similarities and differences between them. Then, we define the concept of power neurons and illustrate an approach to detect them. Next, we demonstrate that power neurons represent a limited set of highly interconnected neurons that form a backbone and that, given their peculiar connectivity properties, may play a strategic role in brain functions. Finally, we extract a set of connectome motifs that allow us to learn about various features characterizing power neurons. We demonstrate that complex network analysis can allow the extraction of relevant knowledge about connectomes. Furthermore, we show that a very small number of power neurons can strongly influence all other neurons in the *Drosophila* brain.

**Supplementary Information:**

The online version contains supplementary material available at 10.1007/s12021-026-09773-6.

## Introduction

Neuronal connectome analysis, i.e. the detailed study of how different brain regions communicate with each other and how brain structure is linked to brain function, is a research field of fundamental importance for understanding how the brain works. This analysis distinguishes between the structural and functional connectomes. The structural connectome refers to the physical connections linking various neuronal elements at the anatomic level, while the functional connectome describes the dynamic interactions between different brain areas and explains how they are operationally connected. Understanding the structure of the connectome allows us to unravel the neural mechanisms underlying complex cognitive functions such as memory, learning, and perception. For example, it has already been shown that specific alterations in the structural connectome (Duda et al., 2025) are associated with neurological disorders such as autism (Lee et al., 2025; Li et al., 2022), epilepsy (Lariviére et al., 2022), Alzheimer’s disease (Calimeri et al., 2021; Fouladi et al., 2022; Fu et al., 2019; Ghanbari et al., 2022) and Parkinson’s disease (Arias-Vergara et al., 2017; Mekyska et al., 2018; Skidmore et al., 2015). Such findings may facilitate the development of new therapeutic strategies based on the modulation (Jahan et al., 2026) of neuronal connections.

There is a close relationship between the structural and functional connectomes; in particular, the former can be seen as the “hardware” of the brain, while the latter can be seen as the corresponding “software”. Based on this analogy, many researchers have contributed to the definition of a new parallel and distributed computing paradigm, called neuromorphic computing (Shrestha et al., 2022), which is inspired by the structure and function of biological neural systems. In this context, many authors aim to bridge the gap between the low-level physical details of biological systems and the higher-level computational functionality. These efforts to emulate biological neural networks have raised expectations for the realization of systems with better noise resilience, greater scalability, and higher energy efficiency. In these research activities, much effort has been devoted to defining the underlying computational models (from both hardware and software perspectives), while little attention has been paid to integrate biological neural networks into these models.

Some recent pioneering work (Lappalainen et al., 2024) used the experimentally determined connectivity of neurons in the motion pathways of the fruit fly optic lobe and optimized the parameters of the resulting neural network using standard deep learning techniques. The model predictions in this work were confirmed by experimental measurements of neural activity over 19 consecutive frames at a frame rate of 24 Hz. This showed that knowing the actual structure i.e. connectivity of neuronal connections can provide powerful insights into simulating the behavior of real organisms. Other recent work has shown the importance of another structural network characteristic: that the distribution of neurons and their connections is not random. For example, in Morales-Gregorio et al. (2023) the authors showed that the density of neurons in the mammalian cerebral cortex follows a log-normal distribution. Furthermore, in Reid and Vempala (2023) the authors showed that geometric random networks – i.e. spatial graphs embedded in a metric space – perform better than Erdos-Renyi random graphs in simulating learning and memorization within the computational model of the brain called NEMO (Papadimitriou et al., 2020; Reid & Vempala, 2023). More generally, in Barabási et al. (2023) the authors emphasized the importance of studying biological neural networks using tools from network analysis. All these results highlight that studying the structural connectomes of real organisms and understanding their features is of great importance and can have a great impact on studying and simulating the behavior of living organisms.

**Fig. 1:**
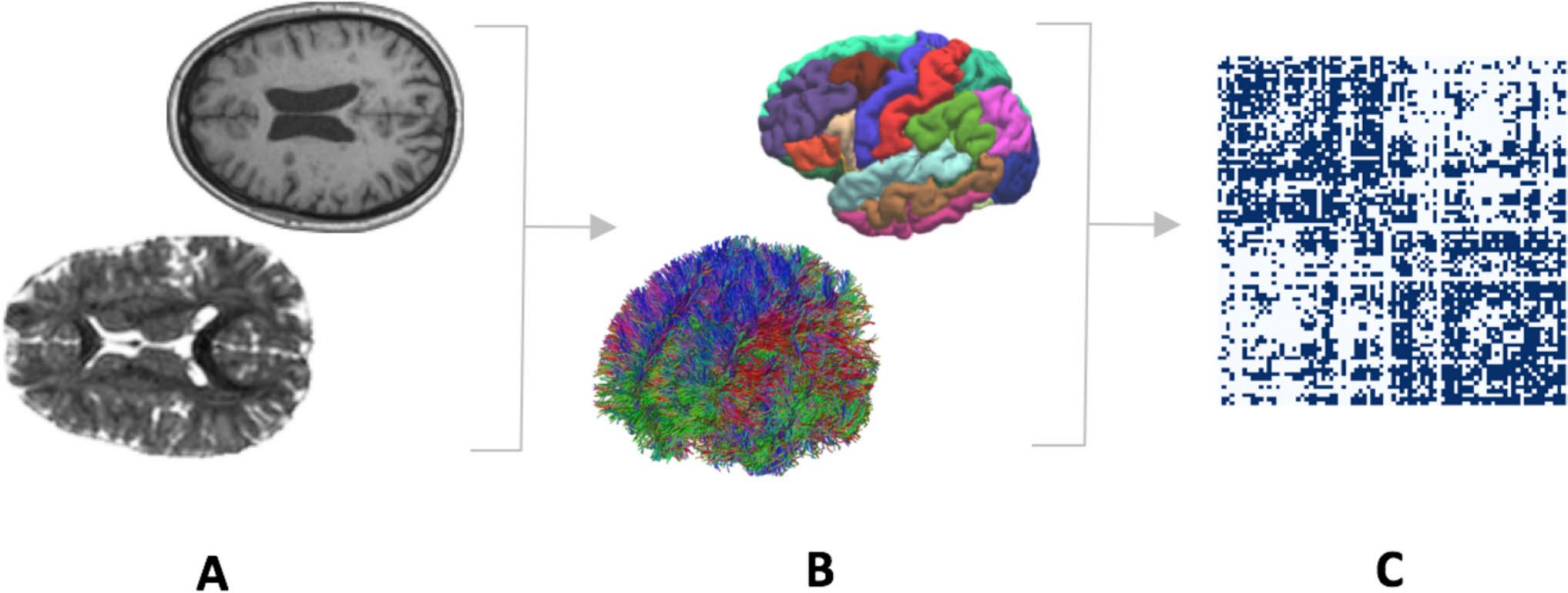
Illustration of the steps for creating a functional connectome: (**A**) T1-weighted and diffusion-weighted MR images are used to generate cortical parcellation and fiber tractography (**B**). These are combined to generate the connectivity matrix (**C**)

Mapping the functional connectome is relatively easy, as it can be obtained using non-invasive methods, such as a combination of Diffusion Magnetic Resonance Imaging (dMRI)-based acquisition methods (like magnetization transfer, spectroscopy, and Diffusion Tensor Imaging - DTI), together with classical structural MRI modalities, like T1. Figure 1 illustrates a pipeline for building functional connectomes from a combination of T1 and DTI (Calimeri et al., 2021). This process includes the following steps: First, a parcellation of cortical and subcortical gray matter (GM) is performed on 3D T1-weighted images to label T1 voxels. Second, diffusion-weighted MRI-images are pre-processed using Eddy-current distortion correction and skull stripping (Jenkinson et al., 2012). Then, probabilistic streamline tractography is applied to the diffusion images to generate fiber tracks in voxels. Finally, the connectivity matrix is generated from the segmented GM brain regions, where connections and their strengths are derived from the number of streamlines connecting them.

In contrast, mapping the structural connectome, and thus the intricate network of physical connections between billions of neurons, is a huge computational undertaking. In particular, current imaging technologies are unable to capture the whole neuronal connectivity at the microscopic level. However, recent research is developing new methods and technologies to address these challenges, including the combined use of ultra-high resolution microscopy, positron emission tomography, and Artificial Intelligence.

These advances have led to groundbreaking discoveries, such as the complete mapping of the connectome of increasingly complex organisms. For example, while the connectome of *Caenorhabditis elegans* was reconstructed as early as 1986 (White et al., 1986), recent advances have made it possible to reconstruct the entire connectome of *Ciona intestinalis* (Ryan et al., 2016) and *Platynereis dumerilii* (Verasztó et al., 2020). At the same time, it has been possible to reconstruct the brain-wide connectivity maps of *Drosophila melanogaster* at different developmental stages (Dorkenwald et al., 2024; Schlegel et al., 2024; Winding et al., 2023). To the best of our knowledge, the complete *Drosophila melanogaster* larval (Winding et al., 2023) and adult (Bates et al., 2025; Berg et al., 2025; Dorkenwald et al., 2024; Schlegel et al., 2024) connectomes represent, to date, the most complex connectomes completely reconstructed and available. Despite its apparent simplicity, *Drosophila melanogaster* has a rich repertoire of interesting behaviors, varying from mating and navigation up to learning and decision-making. Moreover, in terms of research challenges, the availability of the complete connectome of this organism at the larval and adult stages makes it possible to perform developmental analyses and precise comparisons of the connectome between different developmental stages of the same species.

A first application of network analysis to derive some statistics related to the *Drosophila* connectome was performed in (Winding et al., 2023) for the larval stage and in Dorkenwald et al. (2024); Schlegel et al. (2024); Bates et al. (2025); Berg et al. (2025) for the adult stage. In this paper, we aim to go one step further in this direction by analyzing in parallel a type of neurons that characterizes the structure of the *Drosophila* connectome in both larval and adult stages. The knowledge of these neurons allows a better understanding of the complete mechanisms that regulate the behavior of this organism. This approach in *Drosophila* can be seen as a case study that will provide guidelines that can be applied to the analysis of the connectomes of increasingly complex organisms as they become available.

Our research group is investigating several advanced aspects related to *Drosophila* larval and adult connectomes, each of which can give rise to a separate strand, although interconnected with the others. For example, we defined an approach to analyze connectome motifs in larval *Drosophila*. In addition, we are studying the so-called “colonist neurons” in *Drosophila*, i.e. neurons capable of influencing regions of the connectome that are physically distant from their location. Finally, we are investigating the differences that characterize neurons in the two hemispheres of the *Drosophila* brain. Extending our previous larval work (Corradini et al., 2025), this paper advances the state of the art in the study of neuronal connectomes by applying complex network analysis to define and characterize a specific type of neurons, which we call “power neurons”, i.e. neurons of limited number that, given their peculiar connectivity properties, may play a strategic role in brain functions (“Methods” section). In this study, an important role is played by connectome motifs that we had introduced and investigated for larval *Drosophila* in Corradini et al. (2025) and that we extend to adult *Drosophila* in this paper. Although we focused on the *Drosophila* connectome, our approach to detecting and characterizing power neurons is general and can be extended to any connectome.

After introducing the concept of power neurons, in the paper we proceed with an analysis of them from both a theoretical (“Defining Power Neurons” section) and experimental point of view (“Results” section). As for this last task, we first perform an Exploratory Data Analysis of the *Drosophila* larval and adult connectomes (“Exploring Larval and Adult Connectomes” sections); from this analysis, we derive the differences and similarities that characterize these connectomes from a complex network analysis point of view. Specifically, as will be clear below, the average path length and diameter are essentially the same in both connectomes, and the maximum strongly connected component includes nearly all neurons in both of them. The first difference between the two connectomes is scale. Specifically, the adult connectome has a much greater number of neurons and connections than the larval one (two and one orders of magnitude greater, respectively). Additionally, the adult connectome is sparser than the larval one; the density, average node degree, and average clustering coefficient are all lower for the adult connectome than for the larval one. Finally, degree assortativity is moderately positive in the larva, while it is null in the adult. Next, we analyze the distributions of centrality measures on the connectomes of the larva and adult (“Centrality Measures of Larva and Adult Connectomes” section); in fact, as we will see below, centrality measures are the basis of our concept of power neurons, and a detailed analysis of them is essential to better understand the properties of these neurons. Afterwards, we detect power neurons in the connectomes of the larva and adult (“Detecting Power Neurons” section) and then perform a series of analyses to identify their main characteristics (“Characterizing Power Neurons” section). Among these, we will see that power neurons are strategically distributed in the brain and are strongly interconnected, forming a backbone. Finally, we extract a set of connectome motifs centered on power neurons that collectively define a number of important properties characterizing these neurons (“Connectome Motifs Involving Power Neurons” section).

## Methods

### Preliminaries

In this section, we recall some concepts that we have already introduced in Corradini et al. (2025) and that are fundamental to first define and then characterize power neurons, which are the central topic of this paper. These concepts regard modeling neuronal connectomes and defining connectome motifs.

In our research, a neuronal connectome is modeled by a network:

$$ \mathcal {C} = \langle N, A \rangle $$

Here, *N* is the set of nodes in the network. There is a node $n_i \in N$ for each neuron in the connectome. Since there is a biunivocal correspondence between nodes and neurons, we use these two terms interchangeably in this paper. *A* is the set of arcs in the network. There is an arc $(n_i,n_j) \in A$ if there is at least one connection from $n_i$ to $n_j$. We use the terms “connection” and “arc” interchangeably in the following.

In this paper, we use the name “neuronal groups” to uniformly describe groups of homogeneous neurons in the larva (defined as “annotations” (Winding et al., 2023)) and adult (defined as “superclasses” (Dorkenwald et al., 2024; Lin et al., 2024; Schlegel et al., 2024)). As we are not studying the development of the *Drosophila* connectome, i.e. the transition from larva to adult, nomenclature differences across developmental stages do not affect the allocation of power neurons. The two connectomes are compared as two sets of timepoints, larval and adult.

Let $n_i$ be a neuron of $\mathcal {C}$. A connectome motif $CM(n_i)$ of $n_i$ describes to which neuronal group $n_i$ and its connected neurons belong. We also define the in_neighborhood (resp., out_neighborhood) of $n_i$, and denote it by $nbh_{in}(n_i)$ (resp., $nbh_{out}(n_i)$), the set of neurons connected to $n_i$ by arcs incoming in (resp., outgoing from) it.

$$CM(n_i)$$ is a network whose nodes represent neuronal groups and whose arcs represent connections between them. It is obtained from $$n_i$$, $$nbh_{in}(n_i)$$ and $$nbh_{out}(n_i)$$, and the corresponding arcs, by substituting the involved neurons with their respective neuronal groups. Furthermore, if two nodes in $$nbh_{in}(n_i)$$ (resp., $$nbh_{out}(n_i)$$) belong to the same neuronal group, the corresponding nodes in $$ {CM(n_i)}$$ are collapsed into a single node representing that group. $$n_i$$ is defined as the core neuron of $$ {CM}(n_i)$$ and the neuronal group to which $$n_i$$ belongs is defined as the core group of $$ {CM}(n_i)$$. The neuronal groups to which the nodes of $$nbh_{in}(n_i)$$ (resp., $$nbh_{out}(n_i)$$) belong are defined as the input (resp., output) groups of $$CM(n_i)$$. As will become clear in the following, in this paper we are interested in connectome motifs centered on power neurons. Therefore, we will assume that the core neuron $$n_i$$ of a connectome motif $$CM(n_i)$$ considered in this paper is a power neuron.

We first count the number of power neurons from which a connectome motif can be derived, and thus its frequency in the connectome. Next, we perform a Quadratic Assignment Procedure (QAP) test (see below) to test for its statistical significance. Once we have identified statistically significant motifs, we select only those very frequent, i.e. those occurring at least 4 times for the larva and at least 37 times for the adult. Similar to what was done in Lin et al. (2024) for *rich club neurons*, we empirically determined the minimum frequency thresholds (4 for the larva and 37 for the adult) by performing a sensitivity analysis. For each connectome, we explored a range of thresholds and looked for values that guaranteed the identified motifs were robust and did not represent noise, while avoiding the risk of discarding valid motifs as much as possible.

The QAP test is a permutation test suitable for testing hypotheses about quantities that depend on network node labels, while keeping the network structure fixed (Hubert & Schultz, 1976). Since the connectome motifs of neurons are constructed from node labels, the QAP test can be applied to each motif found to test its statistical significance. Specifically, given a connectome motif $$ {CM(n_i)}$$ derived from the network $$\mathcal {C}$$ associated with a *Drosophila* connectome, we want to know whether its number of occurrences is statistically higher than what we would expect from random chance alone. If we were to rewire the network $$ {CM(n_i)}$$ to create a reference null model, we would destroy all features of the network, such as modularity, degree assortativity, distribution over centrality, and so on. Instead, the QAP test works by randomly permuting node labels. By performing the QAP test in our case, it is possible to reassign neuronal groups to nodes, preserving the underlying network structure and creating a reference null model. We permute node labels $$N=1,000$$ times and compute connectome motifs on this set of null models. For each connectome motif $$ {CM(n_i)}$$ found in $$\mathcal {C}$$, we test its statistical significance as follows: let $$\gamma _{ {CM(n_i)}}$$ be the number of times $${CM(n_i)}$$ appears in the original network and let $$\gamma _1, \gamma _2, \ldots , \gamma _N$$ be the number of times it appears in the *N* null models. We calculate the p-value as: $$p_{{CM(n_i)}} = \frac{\sum _{j=1}^N \iota (\gamma _j > \gamma _{{CM(n_i)}})}{N}$$, where $$\iota (\cdot )$$ is the indicator function that returns 1 if the condition in parenthesis is true, 0 otherwise.

### Defining Power Neurons

Our research was made possible by some seminal papers (Bates et al., 2025; Berg et al., 2025; Dorkenwald et al., 2024; Lin et al., 2024; Schlegel et al., 2024; Winding et al., 2023), where the authors completed the first whole-brain connectome of the larval and adult *Drosophila*, and performed analyses including the characterization of node connectivity, the study of simple motifs based on triads and path analysis. Interestingly, the specific investigations conducted in these papers seem complementary in many cases, so that there are various phenomena studied in the larva that have not been studied in the adult, and vice versa. A first goal of our work is to perform a unified analysis of some phenomena capturing similarities and differences across developmental stages.

In Lin et al. (2024), the authors show the existence of a *rich club neurons* in adult *Drosophila* i.e. a subset of neurons that tend to connect with each other more than would be expected by chance alone. In a complex network-based model of the connectome, this would imply the existence of a certain set of nodes characterized by high degree centrality that turn out to be connected more than would be expected by chance alone. In a complex network, the presence of a set of nodes with these characteristics typically results in a better overall network connectivity. Different areas of the network can communicate effectively by exploiting the very connections linking these nodes.

In this paper, we want to go one step beyond the concept of *rich club neurons* and introduce the concept of *power neurons*. These are neurons that, once the connectome is represented by a complex network, correspond to nodes that simultaneously have high values of degree centrality, closeness centrality, betweenness centrality, and eigenvector centrality. In other words, power neurons do not only consider degree centrality, as the *rich club neurons*, but all four basic types of centrality used in network analysis. In this regard, we note that it is well known that, in most contexts that can be modeled by a network, nodes characterized by high values of degree centrality do not have high values of closeness centrality, and vice versa (Tsvetovat & Kouznetsov, 2011). Similar considerations apply to other types of centrality; for example, nodes with high betweenness centrality generally have low degree centrality; similarly, nodes with high eigenvector centrality often have high degree centrality but low closeness centrality (Guimera & Nunes Amaral, 2005; Oldham et al., 2019). The presence of nodes with simultaneously high values of all four types of centrality would be in itself a significant fact. In the following sections, we will show that such a rare occurrence happens for both larval and adult connectomes.

Centralities, a classical concept in network analysis, are at the heart of our definition of power neurons:

- The degree of a node is defined as the number of direct connections it has (Tsvetovat & Kouznetsov, 2011). In the case of directed graphs, one can distinguish between node’s indegree, which represents the number of incoming arcs, and node’s outdegree, which indicates the number of outgoing arcs. According to degree centrality, the higher the degree of a node, the more important it is in the network.
- The closeness of a node is defined as the inverse of its average distance from all other nodes (Tsvetovat & Kouznetsov, 2011), indicating how quickly information can flow from one node to all other nodes, and vice versa. The higher the closeness of a node, the more important it is in the network.
- The betweenness of a node is defined as the sum of the fractions of all-pairs shortest paths that pass through it (Tsvetovat & Kouznetsov, 2011), capturing the ability of a node to act as a bridge between different parts of the network. The higher the betweenness of a node, the more important it is in the network.
- Eigenvector centrality encodes the idea that the importance of a node depends on the number of arcs it has with other nodes and the importance of the latter (Tsvetovat & Kouznetsov, 2011), rendering its definition recursive. Eigenvector centrality is meaningful only for connected networks, hence networks that are not connected should be decomposed into connected components and the eigenvector centrality should be computed for each component separately.

Two other network analysis definitions that we will use in the following are normalized average degree and degree assortativity. Specifically:

- The average degree of a network indicates the average number of direct connections its nodes have and is obtained by calculating the average of the degrees of its nodes. Extending this concept, the normalized average degree of a network is defined as the ratio of the average degree to the number of network nodes. This measure takes into account the size of the network in the comparison of the value of the average degree in different networks, since the same average degree has very different implications for a very large and a very small network.
- Degree assortativity indicates the tendency of nodes in a network to have connections with nodes of similar degree (Newman, 2002). It can be seen as a special case of the more general concept of homophily in network analysis (Khanam et al., 2023). Degree assortativity has value in the real range $[-1,1]$, where positive values are indicators of assortativity, null values are indicators of lack of assortativity, and negative values are indicators of disassortativity, i.e. the tendency of nodes in a network to have connections with nodes of dissimilar degrees.

We are now able to define “power neurons” as those neurons that simultaneously belong to the top 20% sets of neurons characterized by the highest values of degree centrality, closeness centrality, betweenness centrality, and eigenvector centrality.

Power neurons can be characterized as follows:

- They have many connections and can thus act as hubs for other neurons (high degree centrality),
- they are connected to other neurons by medium-to-short paths, so that the information they transmit can reach the neurons of the connectome very quickly (high closeness centrality),
- they are among the few strategic nodes that can carry information between different portions of the connectome (high betweenness centrality),
- and finally they are connected to several other equally central neurons in the network; this allows us to hypothesize the presence of a backbone connecting many of the power neurons (high eigenvector centrality).

Power neurons are very different from the *rich club neurons* proposed in Lin et al. (2024), i.e. neurons with their total degree above a certain threshold set at 37 for adult *Drosophila*, which only consider degree centrality.

## Results

### Datasets

The data used in our research consist of the brain connectome of *Drosophila melanogaster* in two stages, i.e., larval and adult ones.

We extracted the larval connectome following the instructions given in Winding et al. (2023). The data for this connectome are provided as a collection of four adjacency matrices representing the pre-synaptic and post-synaptic connections between dendrites and axons of neurons. Specifically, the four adjacency matrices are as follows: *(i)* the dendrite-dendrite matrix, which represents the connections between the dendrites of the two neurons involved; *(ii)* the axon-dendrite matrix, which represents the connections between the axon of the pre-synaptic neuron and the dendrite of the post-synaptic neuron; *(iii)* the dendrite-axon matrix, which represents the connections between the dendrite of the pre-synaptic neuron and the axon of the post-synaptic neuron; *(iv)* the axon-axon matrix, which represents the connections between the axons of the two neurons involved. In the matrices above, each pair of connected neurons is associated with a weight that represents the number of synapses involved in the connection as a proxy for functional strength. In addition, the dataset provides a further matrix, called ALL-ALL, which merges the four matrices presented above into one.

We extracted the adult connectome following the instructions given in Lin et al. (2024) and companion papers (Dorkenwald et al., 2024; Schlegel et al., 2024). In this dataset, the connections between neurons are represented by the adjacency matrix of a network without distinguishing between different types of connections. This matrix is therefore similar to the larval ALL-ALL matrix. Similar to the larval case, each pair of connected neurons is associated with a weight representing the number of synapses involved in the connection.

Due to the slight differences between the two datasets, and to perform a uniform analysis of the connectome for both larval and adult stages of *Drosophila*, we only used the ALL-ALL matrices as connection type can account for unique synaptic characteristics. This additionally allows us to focus on the presence or absence of a connection between a pair of neurons without taking into account the possible presence of multiple connections between them. Such information would only be available for the larva, making it useless for a uniform study of the larval and adult connectomes. Also, in order not to go too low in abstraction, given a connection between two neurons, we did not consider the number of synapses involved and thus the weight of the connection, but simply its existence.

### Exploring Larval and Adult Connectomes

Larval stage neuronal groups are listed in Table 1. The larva dataset included 346 neurons not associated with any neuronal group (“annotation”). These unclassified neurons were not taken into account when making explicit considerations about neuronal groups. Adult stage neuronal groups are listed in Table 2.

**Table 1:** Larval stage neuronal groups (adapted from Winding et al. (2023)). For each group, the name and description are provided

Neuronal group	Definition
Sensory	Neurons entering the brain from the periphery.
Ascending	Neurons transmitting sensory information from the body to the central brain.
Innate	Neurons belonging to circuits that mediate hardwired, unlearned behaviors, such as the Lateral Horn neurons that mediate innate olfactory behavioral responses.
Learning/memory	Neurons that are part of circuits involved in associative learning and memory formation, storage, and retrieval.
Deep brain	Neurons located entirely within the central brain.
Pre-output	Neurons that are upstream of, and provide direct input to, the final output neurons of the brain.
Brain outputs	Neurons that transmit signals from the central brain to other parts of the body to generate a behavior or physiological change.

**Table 2:** Adult stage neuronal groups (adapted from Schlegel et al. (2024)). For each group, the name and description are provided

Neuronal Group	Definition
Sensory	Neurons that enter the brain from the periphery. Note that “ascending” also includes some sensory neurons, which are not distinguishable from ascending interneurons with any certainty.
Ascending	Neurons that enter the brain from the Ventral Nerve Cord (VNC). These may be sensory or interneurons.
Central	Neurons fully contained in the central brain.
Optic	Neurons fully contained in the optic lobes or ocellar ganglion.
Visual projection	Neurons with dendrites in the optic lobes or ocellar ganglion and axons in the central brain.
Visual centrifugal	Neurons with dendrites in the central brain and axons in the optic lobes or ocellar ganglion.
Descending	Neurons with soma in the brain that exit the brain toward the VNC.
Motor	Neurons that exit the brain toward the periphery (and are therefore considered motor neurons).
Endocrine	Neurons that leave the brain via the Corpora Cardiaca Nerves (NCC) toward the retrocerebral complex (corpora cardiaca and corpora allata).

As a first step in our study, we performed an Exploratory Data Analysis on the larval and adult connectomes to compare some of their basic features considering the theory of complex network analysis (Table 3).

**Table 3:** Values of some of the basic features of the *Drosophila* connectome. For each feature, the values of the larva and adult are provided

Measure	Larva	Adult
Number of nodes (neurons)	2,952	134,181
Number of arcs (connections)	110,677	2,700,513
Average node degree	74.9844	40.2518
Normalized average node degree	0.0254	0.0003
Density	0.0127	0.0001
Average Clustering Coefficient	0.2622	0.1614
Average Path Length	3.28	4.52
Diameter	15	14
Degree Assortativity	0.2515	−0.0557
Maximum (strongly) Connected Component size	2,810	119,756

We unravel the following changes in the connectome of the adult stage compared to the larval stage: *(i)* the number of neurons and connections increases significantly (by two orders of magnitude for neurons and one order of magnitude for connections); *(ii)* the average node degree and the average clustering coefficient decrease; *(iii)* the density decreases significantly (by two orders of magnitude); and *(iv)* the average path length and diameter remain essentially constant. Based on the analysis of these basic features, where the average node degree, density, and clustering coefficient decrease, we hypothesize that growth in connections is less than growth in neurons, i.e. nodes in the adult connectome tend to differentiate more forming more selective connections that aim to link specific neuronal groups with each other. The trend of these basic features observed in *Drosophila* connectomes differs greatly from that observed in other types of networks (e.g., social, technological, and information networks), where density increases and diameter decreases as networks grow (Leskovec et al., 2005).

The degree assortativity is moderately positive in the case of larva and essentially null in the case of adult, whereas the maximum strongly connected components in larval and adult connectomes show very similar behavior. In both cases, we observe the presence of a large maximum (strongly) connected component including 95.19% of the nodes of the larva and 89.25% of the nodes of the adult. This implies that information can easily flow between all or almost all neurons in the larval and adult connectomes.

#### Centrality Measures of Larva and Adult Connectomes

**Fig. 2:**
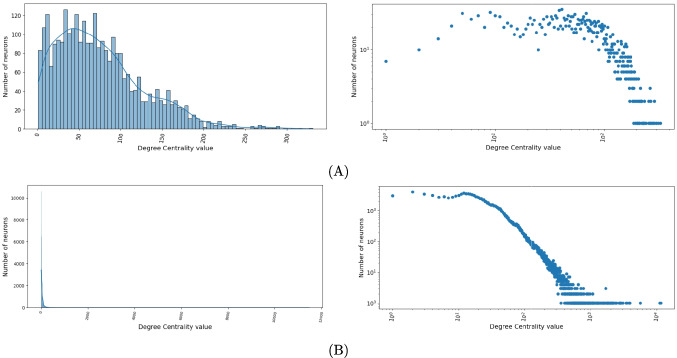
**A** Distribution of degree centrality, in both normal (i) and log-log scales (ii), for the network associated with the larval connectome. **B** As in A for the adult connectome. The degree centrality distribution in the adult follows a steep power law. The larval distribution is a bell-shaped curve superimposed by a second bell-shaped curve, followed by a tail

The degree centrality was the only one used in Lin et al. (2024) to define the *rich club neurons* in adult *Drosophila*. In Fig. 2, we show the distribution of degree centrality, in both normal and log-log scales, for the networks associated with the larval (Fig. 2A) and the adult (Fig. 2B) *Drosophila* connectomes. The distribution of degree centrality in the adult follows a very steep power law, with very few neurons having a very high number of connections and many neurons having a low number of connections. In the larval distribution, we can see the presence of a bell-shaped distribution superimposed by a second bell-shaped distribution and followed by a tail that includes a low number of nodes with very high degree centrality values. This means that in the larva we have many neurons with a medium-low number of connections and few neurons with a high number of connections. Comparing the tails of the larva and adult, we can see that the adult’s tail is much larger, indicating a much greater variability in the number of connections of the neurons of the adult.

**Fig. 3:**
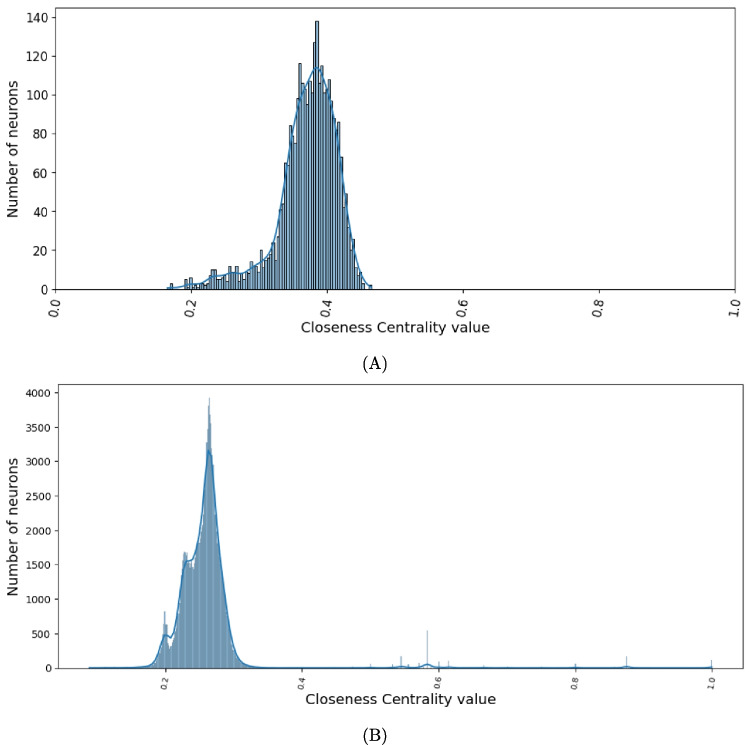
**A** Distribution of closeness centrality for the network associated with the larval connectome. **B** As in A for the adult connectome. Both graphs show that few neurons have a very high closeness centrality, many neurons have a medium closeness centrality, and few neurons have a low closeness centrality

In Fig. 3, we show the distribution of the values of the closeness centrality in the network. Note that in both cases there is a bell-shaped distribution, which is typical for closeness centrality (Tsvetovat & Kouznetsov, 2011). The distribution of the larva is more regular, while that of the adult resembles more the superposition of three bell-shaped curves with gradually increasing heights. Both distributions have a long tail, but the larval distribution is shifted more to the right than the adult one. Both distributions show that few neurons have a very high closeness centrality, many neurons have a medium closeness centrality, and few neurons have a low closeness centrality. Since the closeness centrality of a node in a network is inversely proportional to its average distance from the other nodes in the network (see “Centrality Measures of Larva and Adult Connectomes” section), Fig. 3 tells that, in both the larva and the adult, a small number of neurons are connected to other neurons by short paths, a few are connected by long paths, and the majority are connected by paths that are neither too short nor too long.

**Fig. 4:**
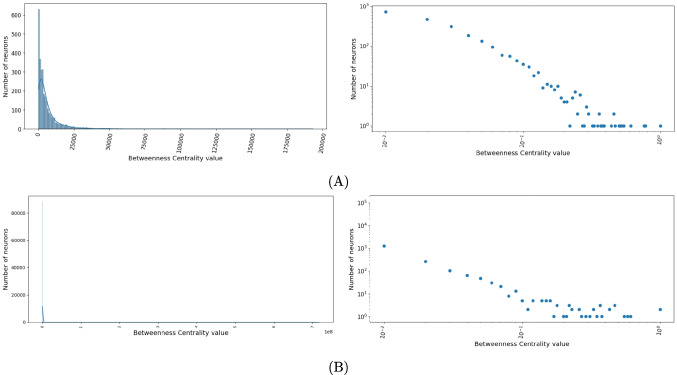
**A** Distribution of betweenness centrality, in both normal (i) and log-log scales (ii), for the network associated with the larval connectome. **B** As in A for the adult connectome. Both distributions follow a power law, with it being steeper and more regular for the adult

The betweenness centrality distributions for the larva and adult are shown in Fig. 4. Both distributions follow a power law, with it being steeper for the adult. The larval distribution has also some irregularities on the left side. The power law distribution characterizing these curves tells us that in the larval and adult connectome, there are many neurons not strategic for connecting different portions of the brain, while there is a small number of neurons very strategic for this task.

**Fig. 5:**
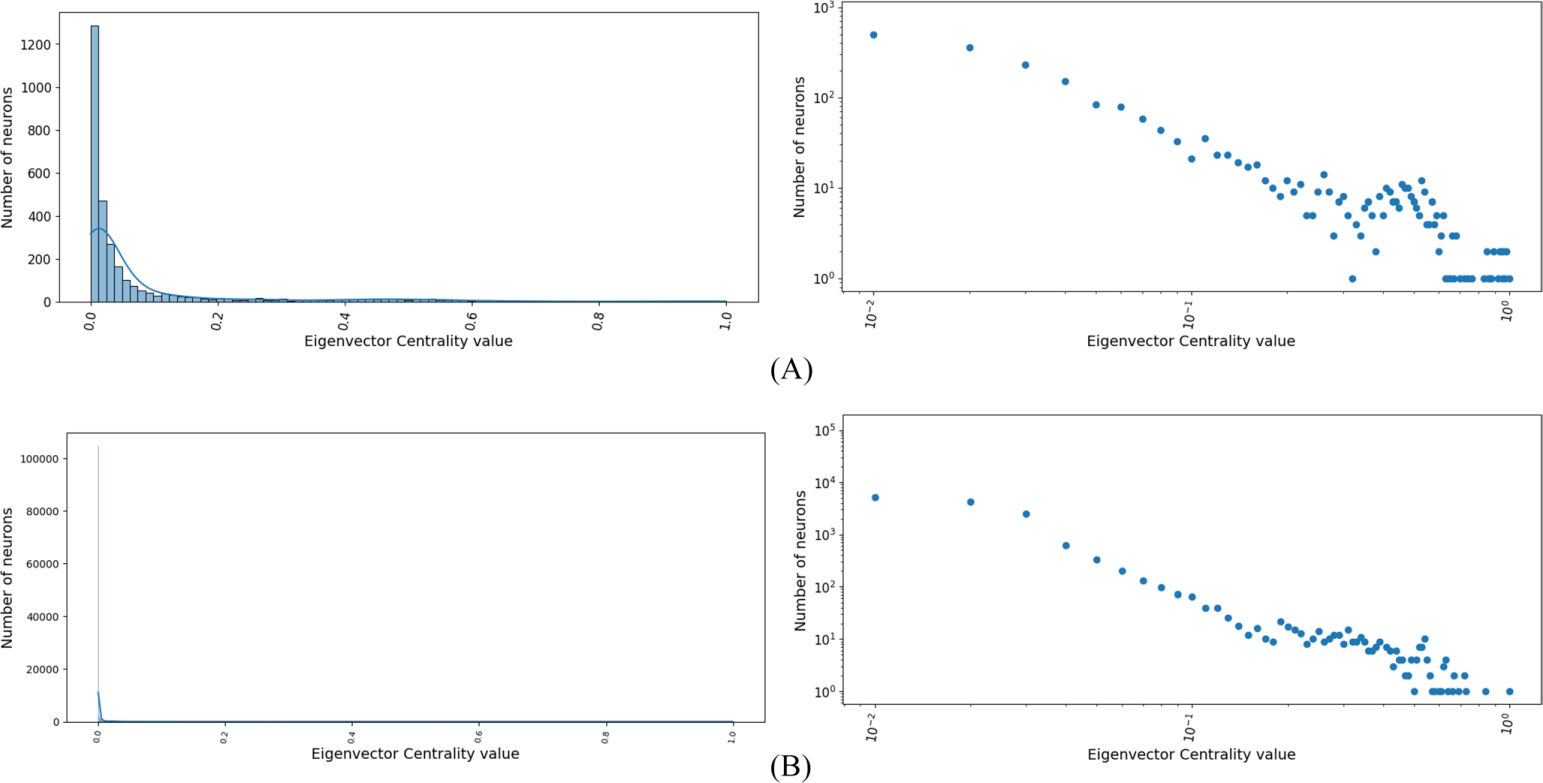
**A** Distribution of eigenvector centrality, in both normal (i) and log-log scales (ii), for the network associated with the larval connectome. **B** As in A for the adult connectome. Both distributions follow a power law, with it being steeper for the adult

The last classical centrality we consider is the eigenvector centrality (Fig. 5). Both distributions follow a power law and as in the previous cases, the adult distribution is steeper than the larval one. Both in the adult and the larval connectomes there are many unimportant neurons because they are linked with few connections to other unimportant neurons. At the same time, there are a few very important neurons because they are linked with many connections to other important or very important neurons.

To complement the visual inspection of Figs. 2–5 and rule out scale effects, we quantitatively compared the centrality distributions of the larva and adult using non-parametric two-sample tests. First, we normalized the degree centrality and betweenness centrality values by dividing each value by their respective maximum possible value. The other centrality values were already normalized. Next, we performed an additional normalization step to convert from the normal scale to the logarithmic scale. To this end, for each value *x* of each centrality, we calculated $\log _{10}(1+x)$. At this point, we applied the Kolmogorov–Smirnov (Table 4) and Mann–Whitney U tests (Table 5) with Benjamini–Hochberg correction to all the resulting centrality values. Both of them confirm that the distributions of centrality values for the larva and adult are very different.

**Table 4:** Values of the statistic (*D*) and p-value for the normalized centrality value distributions of the larva and adult - Kolmogorov-Smirnov test. They confirm that the distributions of centrality values for the larva and adult are very different

Centrality	Statistic (*D*)	p-value
Degree centrality	0.9997	$3.89 \cdot 10^{-242}$
Closeness centrality	0.9774	$1.87 \cdot 10^{-231}$
Betweenness centrality	0.7584	$6.40 \cdot 10^{-167}$
Eigenvector centrality	0.9451	$1.94 \cdot 10^{-216}$

**Table 5:** Values of the statistic (*U*), p-value and Cliff’s delta ($\delta$) for the normalized centrality value distributions of the larva and adult - Mann–Whitney U test. They confirm that the distributions of centrality values for the larva and adult are very different

Centrality	Statistic (*U*)	p-value	Cliff’s delta ($\delta$)
Degree centrality	417	$1.04 \cdot 10^{-183}$	−1.00
Closeness centrality	467,560.5	$1.06 \cdot 10^{-174}$	−0.98
Betweenness centrality	1,369,191.5	$2.86 \cdot 10^{-158}$	−0.93
Eigenvector centrality	210,422.5	$1.12 \cdot 10^{-179}$	−0.99

The presence of a negative $\delta$ with a very high absolute value for degree (resp., closeness, betweenness, eigenvector) centrality indicates that the neurons of the larva have much higher degree (resp., closeness, betweenness, eigenvector) centrality than the neurons of the adult. Median values for each normalized centrality for the larva and adult further corroborate on such differences (Table 6). Consistent with the previous results, when we consider the normalized degrees of neurons in the larva and adult together and take those that exceed the 95th percentile, we see that they correspond to all neurons in the larva and 4.8% of the neurons in the adult.

**Table 6:** Medians for the normalized centrality value distributions of the larva and adult. They confirm that the distributions of centrality values for the larva and adult are very different

Centrality	Median value for the larva	Median value for the adult
Degree centrality	0.0935	0.000164
Closeness centrality	0.4131	0.258563
Betweenness centrality	0.0019	0.000003
Eigenvector centrality	0.1461	0.000048

To exclude that the differences in degree centrality values derive from the adult connectome having a much larger number of neurons compared to the larval one, we performed a subsampling of the adult neurons by randomly selecting 2,952 neurons as the number of neurons present in the larva. We applied the Kolmogorov-Smirnov test to these degree centrality value distributions. We repeated these two tasks (subsampling of adult neurons and application of the Kolmogorov-Smirnov test) 500 times, calculating the mean and standard deviation of the statistic *D*, as well as the corresponding p-value, resulting in a mean value of 0.9997, a standard deviation of 0.0010, and a p-value less than 0.001 in all 500 cases. These results confirmed that the differences in degree centrality distribution between the two connectomes are not an artifact related to the different number of neurons, but rather due to their different characteristics.

In summary, in all types of centrality for both larva and adult we observe the presence of a small number of neurons that have an extremely high centrality value. Could it be that neurons with high centrality values are always the same for all types of centrality?

### Detecting Power Neurons

**Fig. 6:**
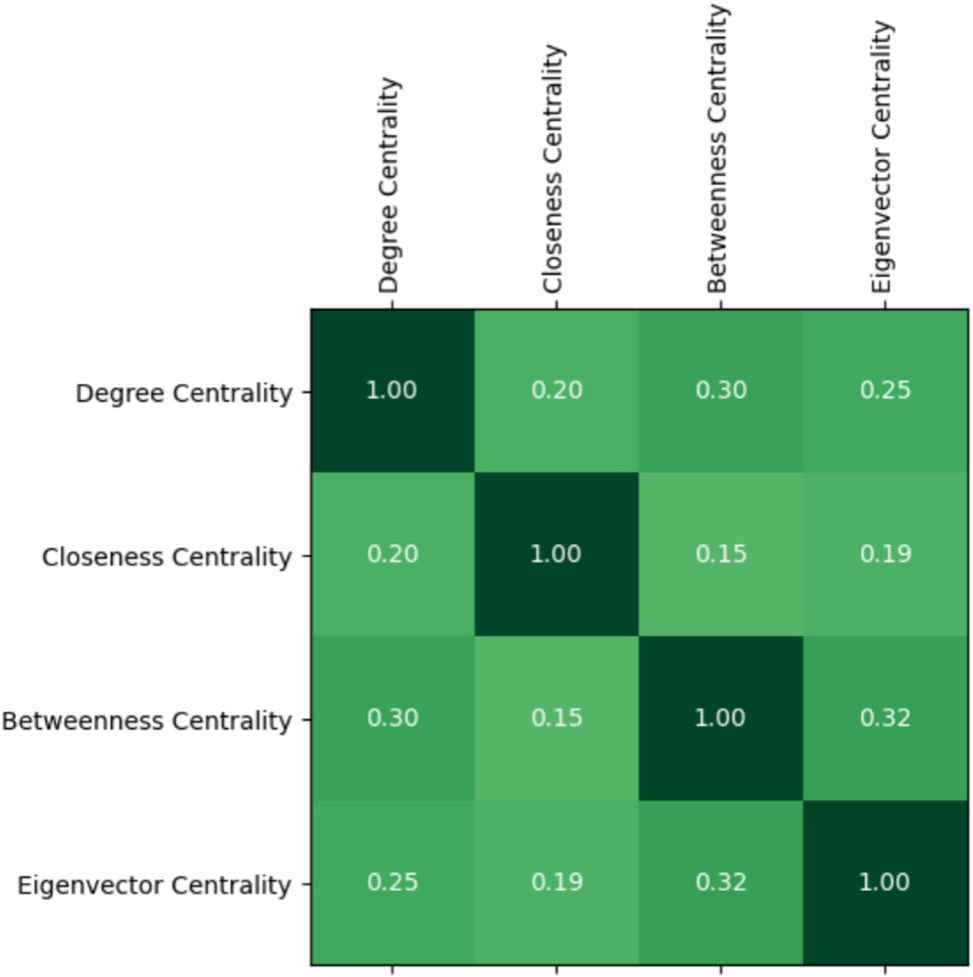
Values of the Spearman’s correlation coefficient for the different pairs of centralities in the larva. Color intensity increases with the magnitude of the coefficient (darker cells indicate stronger correlations)

**Fig. 7:**
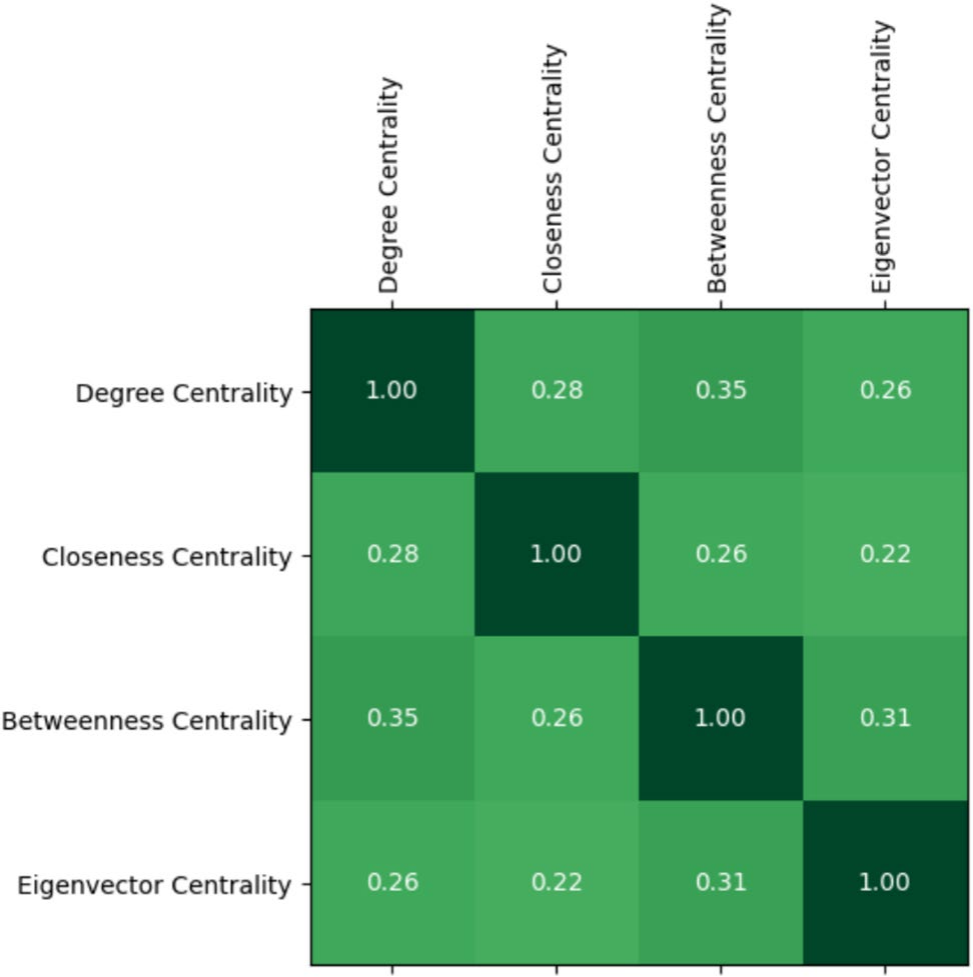
Values of the Spearman’s correlation coefficient for the different pairs of centralities in the adult. Color intensity increases with the magnitude of the coefficient (darker cells indicate stronger correlations)

Next, we checked if there are any correlations between the different forms of centralities both in the larva and in the adult by relying on Spearman’s correlation coefficient (Zar, 2014) (Figs. 6 and 7).

We found positive correlations for all the pairs of centralities considered. What is surprising, however, is that such a positive correlation exists even in cases (e.g., when considering degree and closeness centrality) where network analysis generally does not predict it. This reinforces the hypothesis of the existence of nodes characterized simultaneously by high values for all four centralities.

To test the veracity of this hypothesis, for each centrality distribution in the larva and adult we identified the top 20% of neurons with the highest centrality values. In this way, we obtained eight sets of neurons, four for the larva and four for the adult. The 20% threshold is empirical and is related to the following considerations: *(i)* most distributions follow a power law; *(ii)* we wanted to narrow the focus to the most important neurons for each centrality, but tried to avoid missing some of them. We calculated the intersection between the different pairs of subsets for the larva and adult (Table 7) and found that the percentage of common neurons for each pair of centralities is very high, which, as mentioned before, is generally not the case in the network analysis pointing to the case of *Drosophila* as “special” one. Finally, we calculated the percentage of neurons simultaneously belonging to the top 20% of neurons with the highest values for all four centralities and showed that this percentage is 27.46% for the larva and 20.39% for the adult.

**Table 7:** Percentages of the nodes belonging to the intersection between the top 20% nodes for each pair of centralities. They are very high for each pair of centralities

Centralities	Larva	Adult
Degree & Closeness	62.37%	58.34%
Degree & Betweenness	57.97%	63.78%
Degree & Eigenvector	66.10%	45.86%
Closeness & Betweenness	61.86%	58.48%
Closeness & Eigenvector	51.19%	31.20%
Betweenness & Eigenvector	37.12%	30.53%

In the larva, the fraction of power neurons is $0.2746 \cdot 0.20 = 0.05492$, i.e. the number of power neurons is 162 out of 2,952. Similarly, in the adult, the fraction of power neurons is $0.2039 \cdot 0.20 = 0.04078$, i.e. the number of power neurons is 5,473 out of 134,181. It should be noted that, out of 134,181 neurons in the adult connectome, 60,848 are marked as *rich club neurons*.^1^ Our definition of power neurons allows us to identify a significantly smaller number of neurons (i.e., 5,473).

### Characterizing Power Neurons

In what follows, we characterize the very small set of power neurons on different trajectories and show that they have some very particular characteristics. One of these trajectories refers to the distribution of power neurons between brain hemispheres and, in this respect, we need to refer to the concept of homologous neurons. Two neurons are considered homologous if the structure of their connections is similar but they belong to two different hemispheres. The authors of Winding et al. (2023) explicitly specified which neurons are homologous in the larval dataset. In contrast, the authors of Lin et al. (2024) did not explicitly specify which neurons are homologous in the adult dataset, but provided a variety of attributes, including superclass, subclass, cell types and hemibrain type. From these attributes, we found a way to identify homologous neurons by specifying that they are those neurons having the same values for all neuronal attributes in the dataset, except of course for the attributes root_id (representing the identifier of the neuron) and side (denoting the hemisphere to which the neuron belongs). Regarding the latter attribute, we note that 9 of the 162 power neurons in the larval connectome and 49 of the 5,473 power neurons in the adult connectome are not labeled as “left” or “right”. For this reason, we decided to exclude these neurons from all analyses that involved distinguishing power neurons in the two hemispheres.

#### Power Neurons are Strategically Distributed Among Neuronal Groups in the Brain

By comparing the degree of all neurons and power neurons in the larva and adult, we found that the mean and median degree of power neurons is much higher than that of all neurons (Table 8). This is not surprising, considering that a power neuron is defined as a neuron belonging to the intersection of the top 20% of neurons with the highest degree, closeness, betweenness, and eigenvector centrality values. Consequently, power neurons, by their definition, have a very high degree.

**Table 8:** Mean and median degrees of all neurons and power neurons in the larva and adult. These parameters have much higher values for power neurons than for regular neurons

	Degree		In-Degree		Out-Degree	
	Mean	Median	Mean	Median	Mean	Median
Larva						
All neurons	74.98	65	37.49	32	38.32	32
Power neurons	175.89	165	101.87	97	74.02	71.5
Adult						
All neurons	40.25	22	20.13	9	20.53	12
Power neurons	218.12	152	124.55	86	93.57	60

Interestingly, if we multiply the mean degree of the larval power neurons (175.89) by the number of power neurons (162), we find that there are 28,494 connections in total. For this calculation, we used the mean instead of the median because multiplying the mean number of connections of each neuron by the number of neurons mathematically gives us the total number of connections in the connectome. This property does not hold if we replace the mean with the median, and the difference is particularly high in scenarios with heavy-tailed degree distributions, such as ours. Considering that the larva has 2,952 neurons, we deduce that there is a very high overlap between the sets of neurons directly connected to a given power neuron, averaging to about 10 different power neurons via an incoming or outgoing arc. A similar situation can be found in the adult, where multiplying the mean degree of power neurons (218.12) by the number of power neurons (5,473), we find that the total number of connections is 1,193,770, while the total number of neurons is 134,181. Thus, on average, each neuron in the adult connectome is linked (through an incoming or outgoing arc) to about 9 different power neurons.

**Fig. 8:**
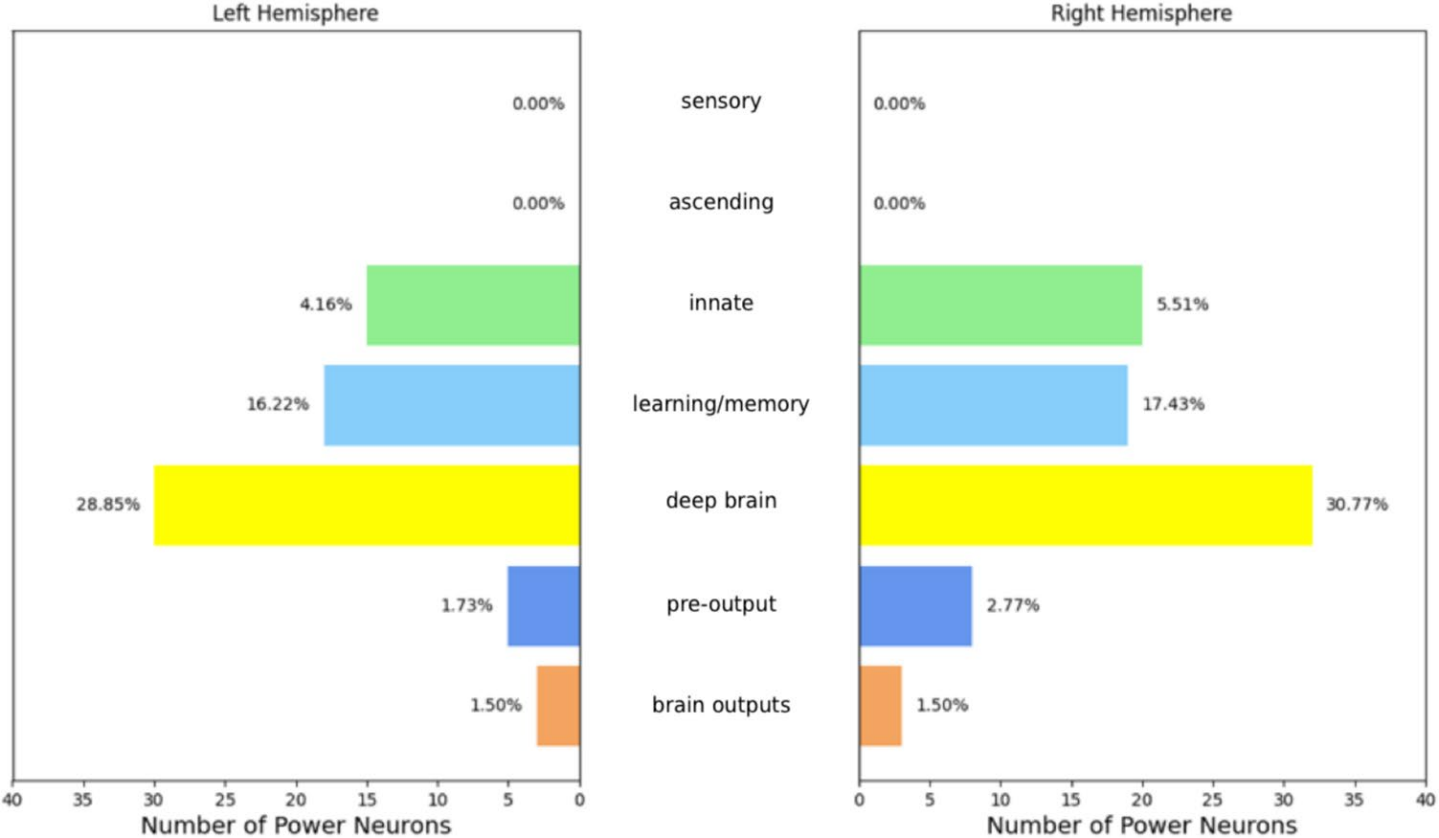
Distribution of the larval power neurons by neuronal group and hemisphere. The length of each bar represents the number of power neurons, while the percentages on the top of the bars indicate the fraction of neurons in the neuronal group/hemisphere that are also power neurons. The “deep brain” and “learning/memory” neuronal groups are significantly over-represented in power neurons

**Fig. 9:**
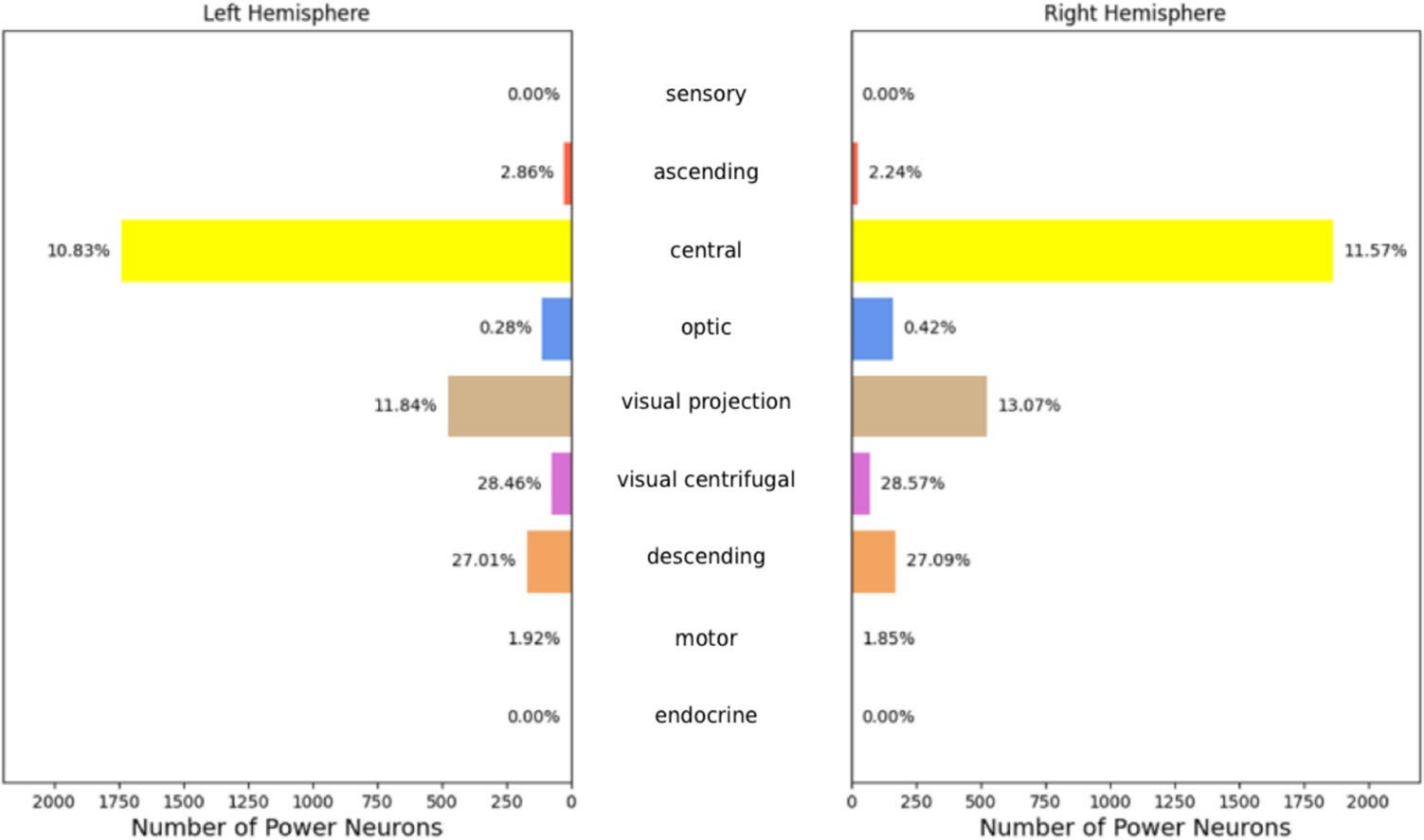
Distribution of the adult power neurons by neuronal group and hemisphere. The length of each bar represents the number of power neurons, while the percentages on the top of the bars indicate the fraction of neurons in the neuronal group/hemisphere that are also power neurons. The “central” and “visual projection” neuronal groups are significantly over-represented in power neurons

The mean indegree (i.e. the number of arcs incoming to a node) and outdegree (i.e. the number of arcs outgoing from a node) for all neurons are substantially equal. Even the median indegree and outdegree are very close. Instead, when we turn to the power neurons, we observe that the mean and median indegrees are always greater than the corresponding outdegrees. This implies that the average number of neurons from which power neurons receive information is greater than the average number of neurons to which they send information. In other words, power neurons are more information receivers than information providers. Considering that most power neurons are located in specific neuronal groups (see “Exploring Larval and Adult Connectomes” section), i.e. “deep brain” and “learning/memory” in the larva (see Fig. 8) and “central” in the adult (see Fig. 9), we can assume that power neurons receive signals from peripheral sensors, process them in the brain, and stimulate the appropriate peripheral sensors based on the processed information.

We now consider the distribution of power neurons in terms of hemispheres. Table 9 summarizes it and characterizes each power neuron on the basis of the following properties: *(i)* hemisphere (right / left) in which it is located; *(ii)* presence or absence of a homolog in the other hemisphere; *(iii)* type (power neuron / simple neuron) of the possible homolog in the other hemisphere. From the analysis of this table we can see that power neurons are basically equally distributed between the two hemispheres in both the larva and adult. In both cases, there is only a slight prevalence of power neurons in the right hemisphere. A large fraction of power neurons have another power neuron as a homolog; this is the case for 73.20% of power neurons in the larva and 73.89% of power neurons in the adult. The rest of the power neurons usually have a homolog, even if it is not a power neuron. This is always the case in the larva, where there is no power neuron without a homolog, and almost always the case in the adult, where only a small percentage of power neurons (i.e., 3.32%) are without homolog.

**Table 9:** Distribution and characterization of power neurons between the hemispheres. Power neurons are basically equally distributed between the two hemispheres in both the larva and adult. There is only a slight prevalence of power neurons in the right hemisphere

	Left hemisphere	Right hemisphere
Larva		
Power neurons	71	82
Power neurons without a homolog	0	0
Power neurons with a homolog	71	82
Power neurons whose homolog is a power neuron	56	56
Power neurons whose homolog is not a power neuron	15	26
Adult		
Power neurons	2,598	2,826
Power neurons without a homolog	77	103
Power neurons with a homolog	2,521	2,723
Power neurons whose homolog is a power neuron	2,004	2,004
Power neurons whose homolog is not a power neuron	517	719

Continuing this analysis, we considered the distribution of power neurons in the different neuronal groups in the two hemispheres. Figures 8 and 9 show both the number of power neurons per neuronal group/hemisphere and the percentage of neurons that are also power neurons in the corresponding neuronal group/hemisphere.

As for the larva, it is easy to see that the “deep brain” and “learning/memory” neuronal groups are significantly over-represented in power neurons. In “deep brain”, about 30% of the neurons are power neurons, while in “learning/memory” the fraction of neurons that are power neurons is about 17%. A significant number of power neurons are also present in the “innate” neuronal group, with a smaller presence of about 5%. A small number and percentage of power neurons are distributed in the “pre-output” and “brain outputs” neuronal groups, while no power neurons are present in the “sensory” and “ascending” neuronal groups. This preliminary analysis shows that different neuronal groups are characterized in very different ways with respect to the presence of power neurons. This prompts us to hypothesize about the different roles of power neurons across brain regions.

As for the adult, the highest number of power neurons is in the “central” neuronal group. However, the highest concentration of power neurons is in the “visual centrifugal” and “descending” neuronal groups, where it is about 28% and 27%, respectively. The “visual projection” neuronal group also contains a significant number and proportion of power neurons (about 12%). In contrast, the “sensory”, “ascending”, “optic”, “motor” and “endocrine” neuronal groups are either unrepresented or under-represented in terms of power neurons. Therefore power neurons are more present and characterizing in certain neuronal groups, potentially accumulating and aggregating information from different brain areas.

**Fig. 10:**
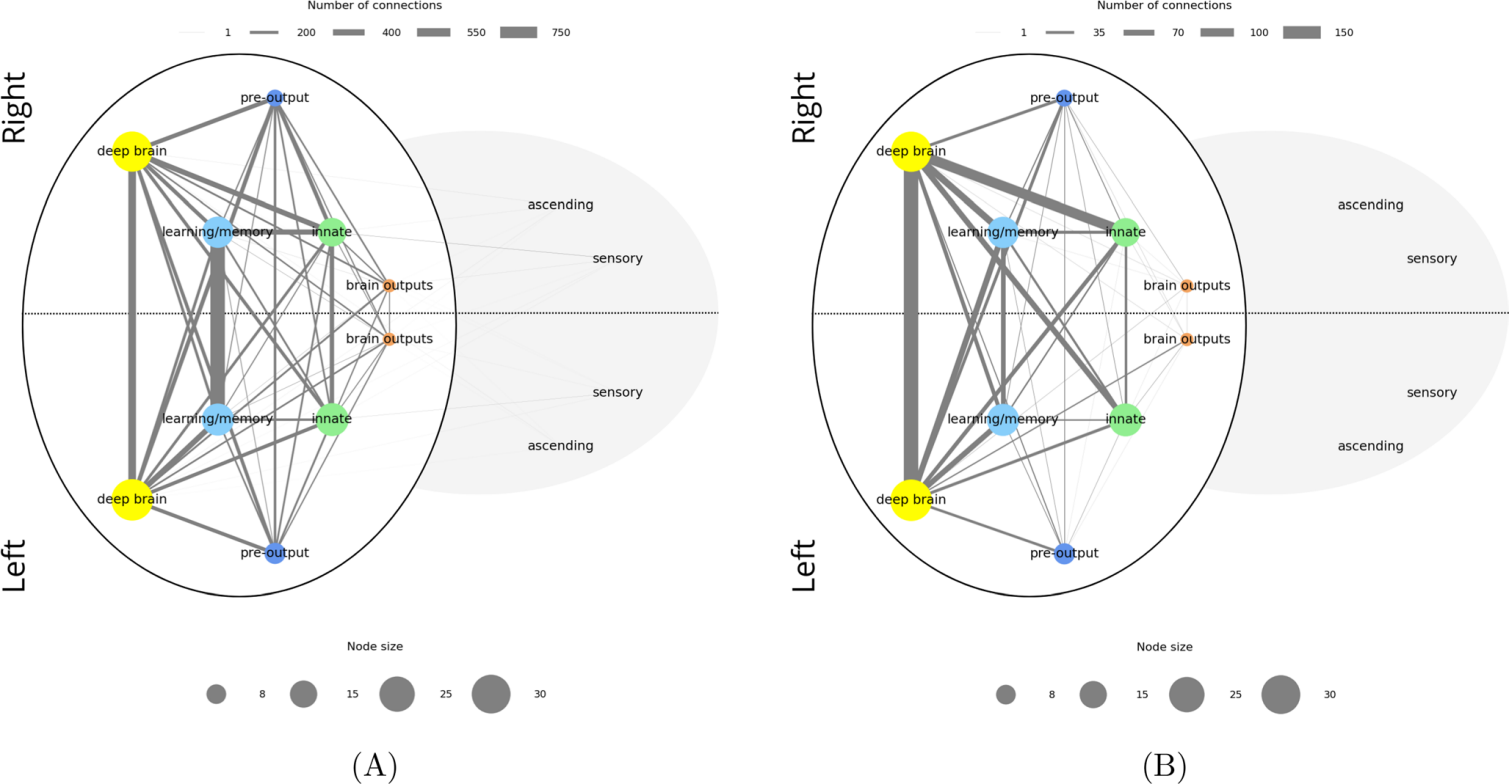
**A** Larval connectome representing intergroup connectivity mediated by power neurons. Each node represents a neuronal group divided by hemisphere (left or right), and the thickness of each node is proportional to the number of power neurons in that group and hemisphere. Edges represent aggregated neuron-to-neuron connections between the corresponding groups. In each connection at least one endpoint neuron is a power neuron. The edge width encodes connection strength, defined as the number of connections between neurons in the two neuronal groups. Legends serve as a visual guide for estimating node sizes and connection numbers. **B** As in **A** but in each connection both endpoint neurons are power neurons

**Fig. 11:**
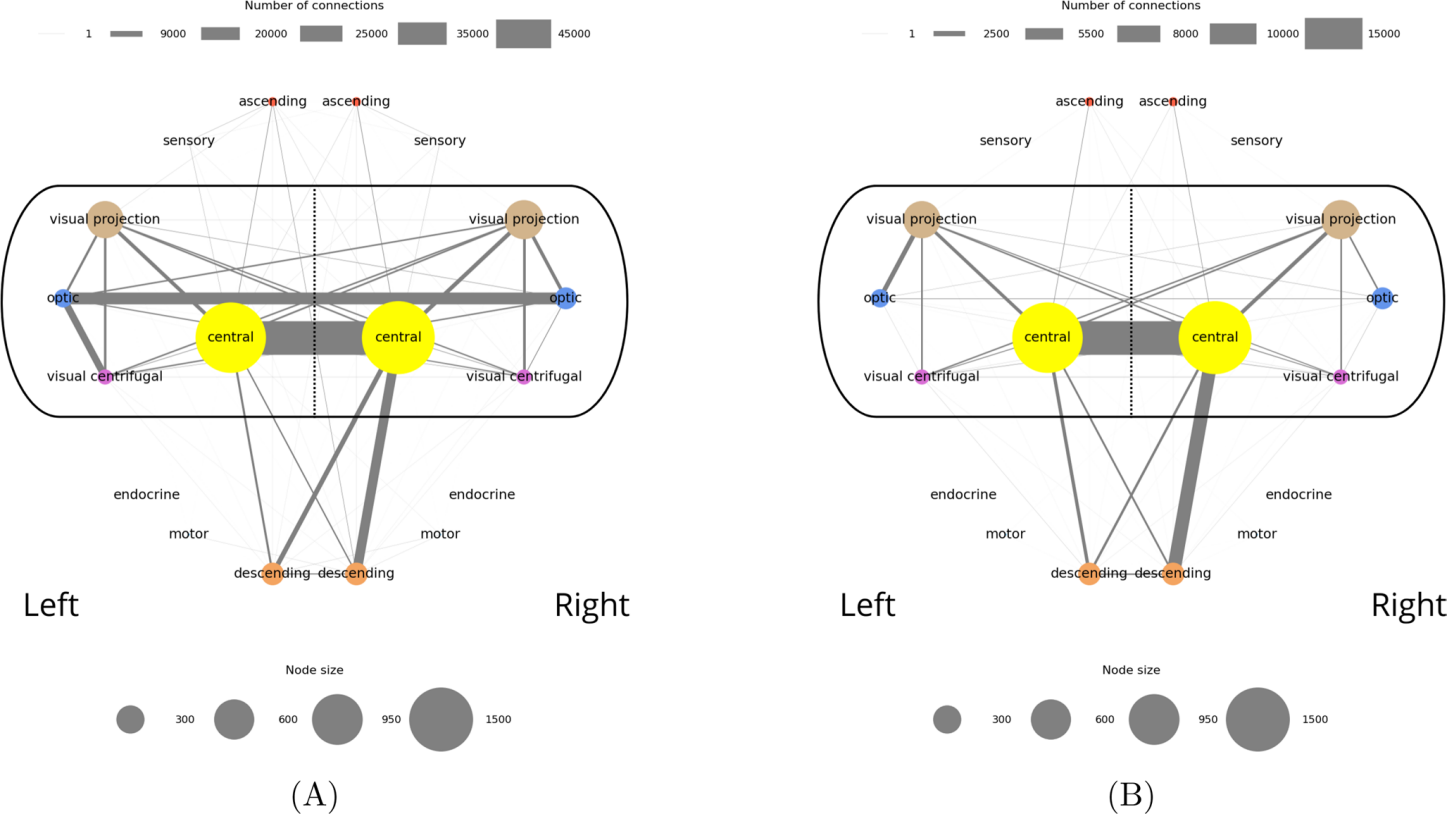
**A** Adult connectome representing intergroup connectivity mediated by power neurons. Each node represents a neuronal group divided by hemisphere (left or right), and the size of each node is proportional to the number of power neurons in that group and hemisphere. Edges represent aggregated neuron-to-neuron connections between the corresponding groups. In each connection at least one endpoint neuron is a power neuron. The edge width encodes connection strength, defined as the number of connections between neurons in the two neuronal groups. Legends serve as a visual guide for estimating node sizes and connection numbers. **B** As in **A** but in each connection both endpoint neurons are power neurons

To address this, we represented power neuron connections in two approaches: In Fig. 10A, a node accounts for each neuronal group and the thickness is proportional to the number of power neurons in that group in the larva. A connection between two nodes exists if at least one of them is a power neuron. In Fig. 10B, connections between two nodes represent connections where both neurons are power neurons. The same applies for the adult (Fig. 11A, B). In both cases, the thickness of the edge is proportional to the number of connections. Extending Figs. 8 and 9, we can now see that the groups with the highest power neuron frequency are the ones that wire together.

Even more interestingly, this also happens between all pairs of neuronal groups in the left and right hemispheres. This confirms the intuition that power neurons are strategic for communication between distant connectome areas. The strongest connections are between left/right partners of the same neuronal groups in “deep brain”, “learning/memory” and “innate”, with “deep brain” and “learning/memory” dominating.

The structure of the graphs in Fig. 10A and B are similar; the most significant but expected change is the complete disconnection of the “ascending” and “sensory” neuronal groups, which not having power neurons could not have connections with the stricter condition characterizing connections in Fig. 10B. It is interesting to note that the connections in the main set still form a complete graph (i.e., a graph in which each pair of nodes is connected by an edge). This implies that, despite their different specializations, all neuronal groups in the main set maintain direct connections with each other. Since these connections involve at least one power neuron (Fig. 10A) or two power neurons (Fig. 10B), we can conclude that this type of neuron plays a fundamental role in ensuring direct communication between the various neuronal groups in the main set. When switching from Fig. 10A to B, the strength of the connections is generally lower, and the number of left/right connections is now greater for the “deep brain” neuronal group instead of the “learning/memory” one. This may be an indicator that power neurons play an even more critical role in the deep brain.

Shifting our analysis to the adult, Fig. 11A shows a less uniform organization than that characterizing the larva. We can still identify a main set of neuronal groups, formed by “central”, “optic”, “visual projection” and “visual centrifugal”, which are more connected than the other ones. In this case, however, this subgraph is not as complete as in the larval case. This means that in the adult, the neuronal groups of the main set are very often directly connected to each other thanks to power neurons. However, compared to the larva, in which all possible pairs of neuronal groups in the main set have a direct connection through one or two power neurons, in the adult there are pairs (for example, the one formed by “visual projection” and “descending”) that are not directly connected to each other through one or two power neurons. Interestingly, the “descending” neuronal group, which is one of the neuronal groups over-represented in terms of power neurons in the adult, is almost exclusively connected to the “central” neuronal group alone, not only by means of intra-hemispheric connections but also through inter-hemispheric ones. Other connections are with the “ascending”, “motor” and “visual centrifugal” neuronal groups. Its counterpart, the “ascending” neuronal group, although under-represented in terms of power neurons, has a similar and symmetric connection structure. In particular, “ascending” neurons are connected to “central”, “descending”, “sensory” and “visual projection” neurons through at least one power neuron. The important property of power neurons to connect distant neurons (e.g., “ascending” and “descending”) is also confirmed in the adult. Note that there is no connection between “optic” and “central” neuronal groups where at least one neuron is a power neuron; in this case, the “visual projection” and “visual centrifugal” neuronal groups seem to act as mediators. In any case, all combinations of possible connections between left / right and “optic” / “visual projection” / “visual centrifugal” neuronal groups via power neurons are present in the resulting graph.

Similar to what we observed for the larva, if we look at the graph with only power neurons (Fig. 11B), the structure of the graph does not change significantly. Clearly, the “motor” and “sensory” neuronal groups are now disconnected, since they have no power neurons. Also, similar to the larva, the connections between the left and right “central” neuronal groups become dominant over the connections between the other neuronal groups.

In summary, we hypothesize that power neurons may play a role in building and maintaining networks of networks within the corresponding connectomes, both larval and adult.

#### Power Neurons Form a Backbone

We have seen that power neurons are a few nodes with characteristics that make them extremely more powerful than other neurons. It is interesting to see if they also form a backbone, that is, if they tend to prefer contacts with each other over contacts with other nodes. To perform this verification, we decided to compute a number of parameters both in the connectome and in the subnetwork induced by the power neurons, that is, the subnetwork consisting only of the power neurons and the connections between them. The parameters we measured are number of nodes, number of arcs, density, average clustering coefficient, diameter, average shortest path, size of the maximum connected component, average degree, normalized average degree, and degree assortativity.

In Table 10, we report the parameter values obtained for the complete connectome and for the subnetwork of the connectome induced by power neurons alone for both the larva and the adult.

**Table 10:** Main characteristics and measures of the complete network mapping the connectome and the power neuron subnetwork

	Complete network mapping the connectome	Subnetwork induced by power neurons
Larva		
Number of nodes	2,952	162
Number of arcs	110,677	3,224
Density	0.0127	0.1236
Average clustering coefficient	0.2622	0.3591
Diameter	15	5
Average shortest path length	3.28	2.18
Maximum connected component’s size	2,810	162
Average node degree	74.9844	39.8025
Normalized average degree	0.0254	0.2457
Degree Assortativity	0.2515	0.2012
Adult		
Number of nodes	134,181	5,473
Number of arcs	2,700,513	223,924
Density	0.0001	0.0074
Average clustering coefficient	0.1614	0.1734
Diameter	14	7
Average shortest path length	4.52	2.96
Maximum connected component’s size	119,756	5,468
Average node degree	40.2518	81.8286
Normalized average degree	0.0003	0.0150
Degree Assortativity	−0.0557	−0.0917

Indeed: The density in the induced subnetwork is much higher than in the whole network (specifically, it is 9.73 times higher in the larva and 74 times higher in the adult). The maximum strongly connected component includes a higher fraction of nodes in the induced subnetwork than in the complete network; specifically, for the larva, it includes 100% of the nodes in the induced subnetwork and 95.19% of the nodes in the complete network; for the adult, it includes 99.90% of the nodes in the induced subnetwork and 89.25% of the nodes in the complete network. The average clustering coefficient of the induced subnetwork is higher than in the complete network in both the larva and the adult. The normalized average degree in the induced subnetwork is much higher than in the complete network (in particular, it is about 9.67 times higher in the larva and 50 times higher in the adult). The average shortest path in the induced subnetwork is smaller than that in the complete network, for both the larva and the adult.

A final interesting insight we can derive concerns assortativity. Indeed, we can observe that: In the case of the larva, there is significant assortativity in both the complete network and the induced subnetwork. The assortativity in the complete network indicates that high degree nodes tend to connect with other high degree nodes, and vice versa. Actually, the existence of this phenomenon could already be inferred indirectly from the analyses performed in the previous sections. What is really surprising is that assortativity persists even in the induced subnetwork. Since the latter contains only power neurons, which, as we have seen, generally have high degrees, the persistence of degree assortativity in this network indicates that there might exist a hierarchy among power neurons. In the case of the adult, assortativity is essentially null in both the induced subnetwork and the complete network, and no further insights can be derived.

#### Connectome Motifs Involving Power Neurons

In this experiment, we wanted to see if there were any connectome motifs (see “Preliminaries” section) in which the core neuron was a power neuron. An example of such a motif is shown in the first row of Table 12 and graphically illustrated in the first network of Fig. 14. It indicates that there is a subnetwork involving neurons from very specific neuronal groups; in particular, there are 202 power neurons of the “central” neuronal group connected by incoming arcs to neurons of the “ascending”, “central”, and “visual projection” neuronal groups, and by outgoing arcs to neurons of the “central” and “descending” neuronal groups. The “central” neuronal group is the core group of this motif because the power neurons from which it originates belong to it. The count equal to 202 means that this motif occurs 202 times in the connectome. In other words, we are saying that this connectome motif, with power neurons from a very specific core group (i.e., “central”), is very common in the adult connectome because it occurs 202 times, each time with a different power neuron from the “central” core group.

To avoid selecting connectome motifs whose frequency is due to chance, we verified their statistical significance by means of the QAP test (see “Preliminaries” section).

**Fig. 12:**
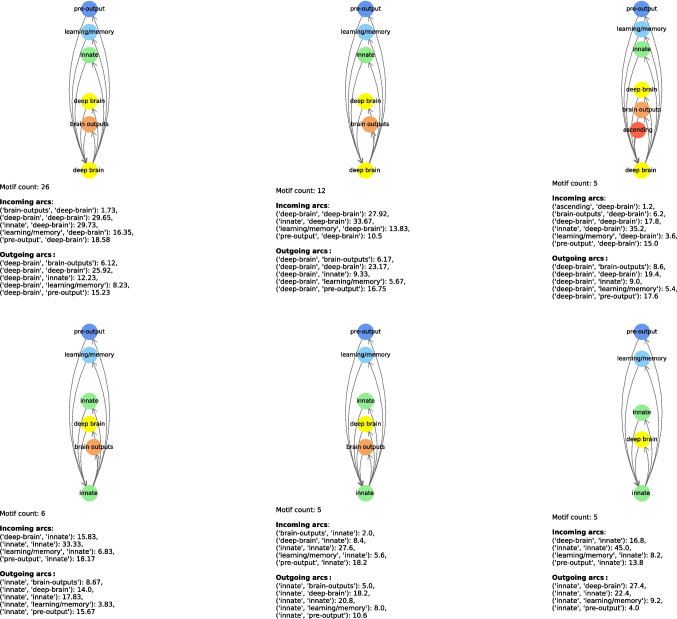
Graphical representation of most frequent connectome motifs in the larva with “deep brain” (i) and “innate” (ii) as core group. In each diagram, the bottom node represents the core group, while the upper nodes represent the input and output neuronal groups. Incoming (resp., outgoing) arcs represent connections from (resp., to) the core power neuron. The reported “Motif count” is the number of occurrences of the same motif in the connectome. The values listed under “Incoming arcs” and “Outgoing arcs” report the average number of connections per motif occurrence for each pair of neuronal groups involved

**Fig. 13:**
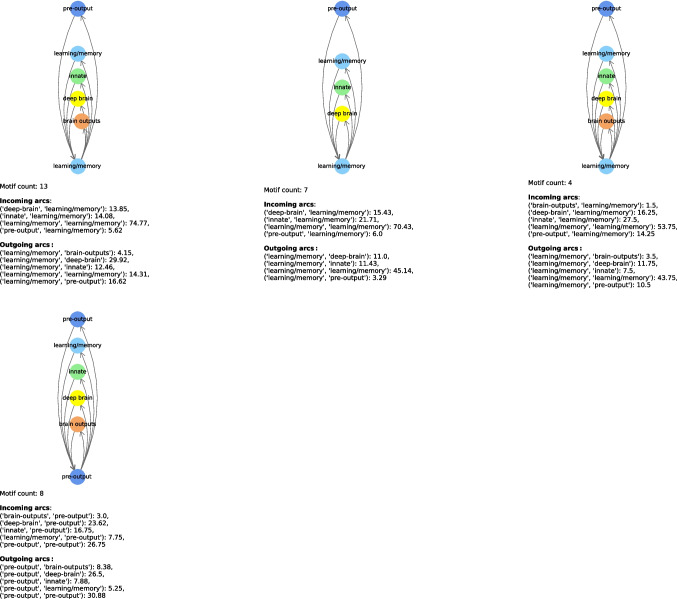
Graphical representation of most frequent connectome motifs in the larva with “learning/memory” (i) and “pre-output”(ii) as core group. In each diagram, the bottom node represents the core group, while the upper nodes represent the input and output neuronal groups. Incoming (resp., outgoing) arcs represent connections from (resp., to) the core power neuron. The reported “Motif count” is the number of occurrences of the same motif in the connectome. The values listed under “Incoming arcs” and “Outgoing arcs” report the average number of connections per motif occurrence for each pair of neuronal groups involved

**Fig. 14:**
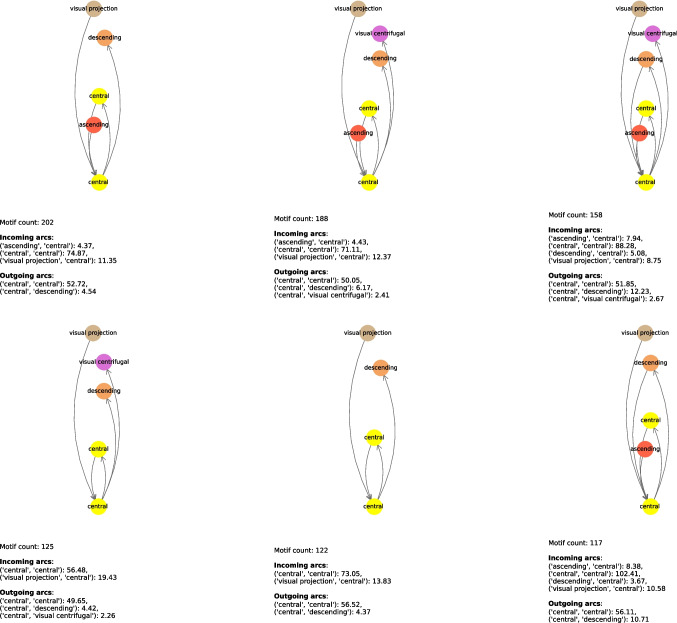
Graphical representation of the most frequent connectome motifs by core group in the adult with “central” as the core group. In each diagram, the bottom node represents the core group, while the upper nodes represent the input and output neuronal groups. Incoming (resp., outgoing) arcs represent connections from (resp., to) the core power neuron. The reported “Motif count” is the number of occurrences of the same motif in the connectome. The values listed under “Incoming arcs” and “Outgoing arcs” report the average number of connections per motif occurrence for each pair of neuronal groups involved

**Fig. 15:**
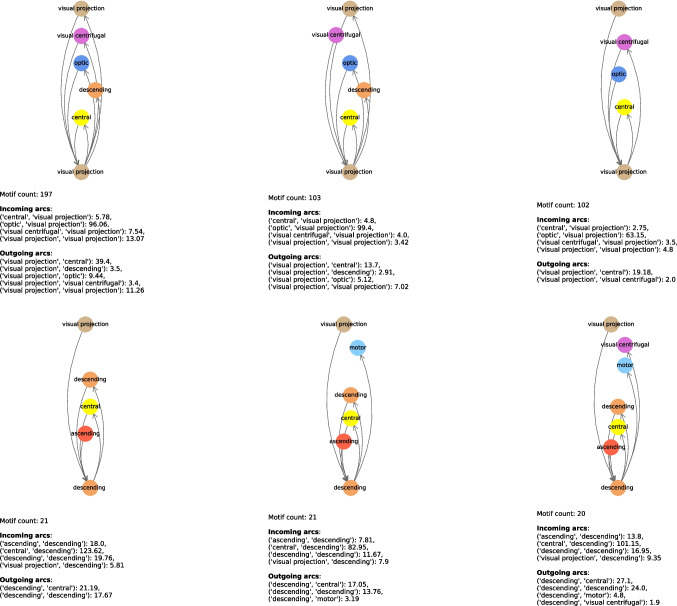
Graphical representation of the most frequent connectome motifs by core group in the adult with “visual projection” (i) and “descending”(ii) as core group. In each diagram, the bottom node represents the core group, while the upper nodes represent the input and output neuronal groups. Incoming (resp., outgoing) arcs represent connections from (resp., to) the core power neuron. The reported “Motif count” is the number of occurrences of the same motif in the connectome. The values listed under “Incoming arcs” and “Outgoing arcs” report the average number of connections per motif occurrence for each pair of neuronal groups involved

**Fig. 16:**
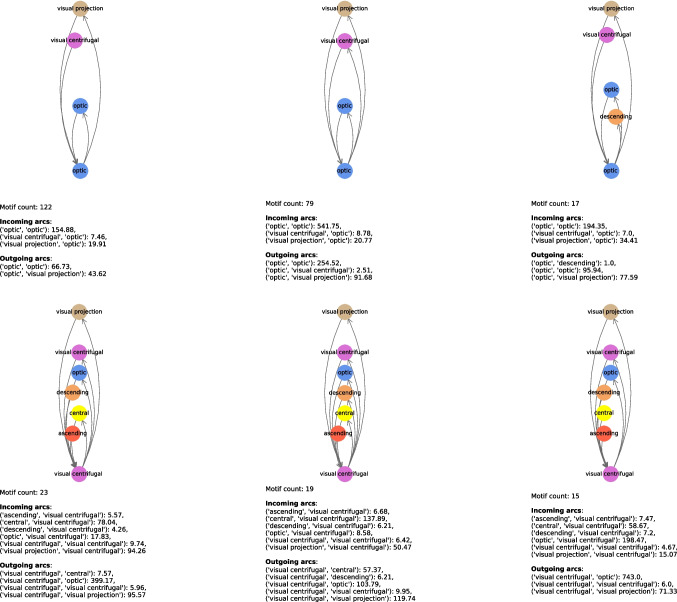
Graphical representation of the most frequent connectome motifs by core group in the adult with “optic” (i) and “centrifugal” (ii) as core group. In each diagram, the bottom node represents the core group, while the upper nodes represent the input and output neuronal groups. Incoming (resp., outgoing) arcs represent connections from (resp., to) the core power neuron. The reported “Motif count” is the number of occurrences of the same motif in the connectome. The values listed under “Incoming arcs” and “Outgoing arcs” report the average number of connections per motif occurrence for each pair of neuronal groups involved

In Tables 11 and 12, we show the most relevant connectome motifs in the larva and adult respectively. For each motif, we report: *(i)* the core group, *(ii)* the input groups, *(iii)* the output groups, and *(iv)* the number of occurrences of that motif. All connectome motifs shown in the tables passed the QAP test and are therefore statistically significant.^2^ In addition, in Figs. 12, 13, 14, 15 and 16 we provide a graphical representation of some motifs, grouped by their core group. In these figures, the bottom node represents the core group, the top nodes represent the input and output groups, and the arcs incoming into and outgoing from the core group represent the connections between the neurons in the input and output groups and the core power neurons. Below each motif, for each type of arc incoming in or outgoing from the core group and for each pair of neuronal groups, we report the average number of connections of that type for that pair. For example, in the first connectome motif in Fig. 12, the average number of connections from “innate” to “deep brain” (the latter being the core group for this motif) is 29.73, meaning that for each occurrence of the motif, and thus for each core power neuron of “deep brain”, there were an average of 29.73 neurons of “innate” that had a connection to the core power neuron and were merged into a single node associated with “innate” when the connectome motif was constructed. Note that this number is completely independent of the *Count* value of this motif, which is 26, and thus independent of the number of occurrences of the motif. In fact, the latter indicates how many power neurons in the core group share the same configuration of input/output groups. In contrast, the number of connections between a neuronal group and the core group refers to a single occurrence of a motif, and thus to a single core power neuron.

The motifs in Figs. 12–16 were selected not based on occurrences, but based on the fact they had all possible core groups.

**Table 11:** Most frequent connectome motifs in the larva. For each motif, the *Input* column lists the input neuronal groups. The *Output* column lists the output neuronal groups. The *Core group* column identifies the neuronal group of the core power neuron. The *Count* column reports the number of occurrences of the motif in the connectome

Input	Core group	Output	Count
Brain outputs, deep brain, innate,	Deep brain	brain outputs, deep brain, innate,	26
learning/memory, pre-output		learning/memory, pre-output	
Deep brain, innate, learning/memory,	Learning/memory	Brain outputs, deep brain, innate,	13
pre-output		learning/memory, pre-output	
Deep brain, innate, learning/memory,	Deep brain	Brain outputs, deep brain, innate,	12
pre-output		learning/memory, pre-output	
Brain outputs, deep brain, innate,	Pre-output	Brain outputs, deep brain, innate,	8
learning/memory, pre-output		learning/memory, pre-output	
Deep brain, innate, learning/memory,	Learning/memory	Deep brain, innate, learning/memory,	7
pre-output		pre-output	
Deep brain, innate, learning/memory,	Innate	Brain outputs, deep brain, innate,	6
pre-output		learning/memory, pre-output	
Brain outputs, deep brain, innate,	Innate	Brain outputs, deep brain, innate,	5
learning/memory, pre-output		learning/memory, pre-output	
Deep brain, innate, learning/memory,	Innate	Deep brain, innate, learning/memory,	5
pre-output		pre-output	
Ascending, brain outputs, deep brain,	Deep brain	Brain outputs, deep brain, innate,	5
innate, learning/memory, pre-output		learning/memory, pre-output	
Brain outputs, deep brain, innate,	Learning/memory	Brain outputs, deep brain, innate,	4
learning/memory, pre-output		learning/memory, pre-output

**Table 12:** Most frequent connectome motifs in the adult. For each motif, the *Input* column lists the input neuronal groups. The *Output* column lists the output neuronal groups. The *Core group* column identifies the neuronal group of the core power neuron. The *Count* column reports the number of occurrences of the motif in the connectome

Input	Core group	Output	Count
Ascending, central, visual projection	Central	Central, descending	202
Central, optic, visual centrifugal,	Visual projection	Central descending, optic,	197
visual projection		visual centrifugal, visual projection	
Ascending, central, visual projection	Central	Central, descending, visual centrifugal	188
Ascending, central, descending, visual projection	Central	Central, descending, visual centrifugal	158
Central, visual projection	Central	Central, descending, visual centrifugal	125
Central, visual projection	Central	Central, descending	122
Optic, visual centrifugal, visual projection	Optic	Optic, visual projection	122
Ascending, central, descending,	Central	Central, descending	117
visual projection			
Central, visual projection	Central	Central	106
Central, optic, visual centrifugal,	Visual projection	Central, descending, optic,	103
visual projection		visual projection	
Central, optic, visual centrifugal,	Visual projection	Central, visual centrifugal	102
visual projection			
Ascending, central, visual projection	Central	Central	89
Central, optic, visual centrifugal, visual projection	Visual projection	Central, visual centrifugal, visual projection	85
Ascending, central, descending, visual centrifugal,	Central	Central, descending, visual centrifugal,	81
visual centrifugal, visual projection		visual projection	
Central, optic, visual centrifugal,	Visual projection	Central, descending, visual centrifugal,	80
visual projection		visual projection	
Ascending, central, visual projection	Central	Central, descending, visual centrifugal	80
		visual projection	
Optic, visual centrifugal, visual projection	Optic	Optic, visual centrifugal, visual projection	79
Ascending, central, descending,	Central	Central, descending, visual centrifugal,	70
visual projection		visual projection	
Central, optic, visual centrifugal,	Visual projection	Central, optic, visual centrifugal,	69
visual projection		visual projection	
Ascending, central, descending,	Central	Central, descending, visual centrifugal	67
visual centrifugal, visual projection			
Central, visual projection	Central	Central, visual projection	61
Ascending, central, visual centrifugal,	Central	Central, descending, visual centrifugal,	58
visual projection			
Central, visual projection	Central	Central, visual centrifugal	58
Ascending, central, descending	Central	Central, descending, visual centrifugal	51
Ascending, central, visual projection	Central	Central, visual projection	50
Central, visual projection	Central	Central, descending, visual centrifugal	48
		visual projection	
Ascending, central, visual centrifugal,	Central	Central, descending, visual centrifugal,	45
visual projection		visual projection	
Ascending, central, descending	Central	Central, descending	45
Central	Central	Central, descending, visual centrifugal	44
Central, optic, visual centrifugal, visual projection	Visual projection	Central, optic, visual projection	43
Ascending, central, visual projection	Central	Central, descending, visual projection	43
Ascending, central, descending, visual projection	Central	Ascending, central, descending, visual centrifugal	43
Central	Central	Central, descending	38
Central, visual projection	Central	Central, descending, visual projection	37

Table 11 shows that connectome motif counts for the larva are not very high. It also shows that the neuronal groups most represented in the power neurons, namely “deep brain”, “learning/memory” and “innate”, are also the core groups for the most frequent motifs. We observe that almost all power neurons in the “pre-output” neuronal group are organized in the same motif, which actually collects all neuronal groups except “sensory” and “ascending”. Actually, “sensory” and “ascending” are almost completely unrepresented in the most frequent connectome motifs of the larva. In fact, not only these neuronal groups have no power neurons, and thus cannot be core groups for any motif, but they also appear only occasionally as input and output groups in the connectome motifs found. Their absence from these motifs does not imply that they cannot participate in generic frequent structures; they are simply not directly connected to power neurons through frequent motifs. In conclusion, these two neuronal groups have specific characteristics that are quite different from those of the other neuronal groups.

Additionally, the most frequent connectome motifs are strongly centered on the core group, having both input and output arcs from and to it. Note that this feature does not necessarily imply the presence of loops between neurons; in fact, different neurons and power neurons from the same neuronal group could be connected by these arcs. Observe also that two occurrences of the same connectome motif have different core power neurons originating them. For example, there are 8 different occurrences of the motif in Fig. 13ii. This is possible because, as mentioned above, multiple neurons and power neurons may belong to the same neuronal group. In the case of the motif in question, there are exactly 8 different power neurons belonging to the “pre-output” core group with the same configuration of input and output groups. The 8 occurrences related to this motif are generated by 8 different power neurons of the “pre-output” core group.

Looking again at the connectome motifs of the larva, we can see that the structures of the most frequent connectome motifs are very similar to each other, both in the case where we consider connectome motifs with the same core group, and in the case where we consider connectome motifs with different core groups (Figs. 12 and 13). For example, consider the first row of Fig. 12, which represents the three most frequent connectome motifs with “deep brain” as the core group. The difference between the first and second connectome motifs is only the absence of an arc from “brain outputs” to “deep brain”. However, note that the average number of connections from “brain outputs” to “deep brain” is only 1.73, which is a very low value; consequently, these two connectome motifs can be considered basically equivalent. Similarly, in the same row, the only difference between the first and the third connectome motif is the presence of an incoming arc from the “ascending” neuronal group, and again the average number of connections between “ascending” and “deep brain” is very low, equal to 1.2. Again, we can consider these two connectome motifs as basically equivalent. Now, let us consider the first connectome motif with “deep brain” as the core group (i.e. the first connectome motif of Fig. 12i) and the second connectome motif with “innate” as the core group (i.e. the second connectome motif of Fig. 12ii). They are structurally identical the only difference being the corresponding core groups.

Analyzing Figs. 12 and 13, we can see that the average number of connections to core power neurons is generally higher for neurons belonging to the core group than for neurons belonging to different neuronal groups. In addition, we observe that most of the connectome motifs involve all or most of the neuronal groups. As a further feature, the set of input groups in each larval connectome motif is often the same as the set of output groups. Therefore, we can say that there is often a symmetry between input and output groups in the larval connectome motifs. Consider, for example, the first larval connectome motif shown in Table 11. Its input and output groups are exactly the same. This is true for all other connectome motifs of the table except the second, third and sixth.

Turning to the connectome motif analysis for the adult, the first observation we can make is that these motifs are much more heterogeneous than the ones of the larva. In fact, in the adult, the input and output groups are rather heterogeneous, and this is true both when considering connectome motifs having the same core groups, and when considering connectome motifs having different core groups. For example, consider the first, fourth, seventh and last larval connectome motifs, shown in Table 11. These motifs have different core groups, but the same input and output groups. The second and third motifs in the same table are another example; indeed, they have different core groups, but the same input and output groups. Furthermore, the sixth and seventh motifs have the same core group and output group, the only difference being the presence of an additional neuronal group in the input groups of the seventh motif. This level of homogeneity is not found in the adult (Table 12), where motifs sharing the same input or output groups are much rarer. Unlike what happens in the larva, the sets of input and output groups in each adult connectome motif significantly differ. Therefore, there is generally no symmetry between input and output groups in adults. In fact, very few motifs in Table 12 share the same sets of input and output groups; in particular, this occurs in only 4 out of 34 motifs (e.g., the 17th motif).

The number of neuronal groups considered is comparable in the two organisms (in fact, it is 7 in the larva and 9 in the adult). Similarly to the larva, in the adult we observe that the average number of connections to the core power neuron is higher for neurons belonging to the core group than for neurons belonging to different neuronal groups.

Many common connectome motifs have “central” as their core group. This is not surprising given the very large number of power neurons in this neuronal group. We can also observe that there are connectome motifs involving only a few neuronal groups. For example, motifs whose core power neurons are located in the “optic” neuronal group involve only the “visual centrifugal”, “visual projection” and occasionally “descending” neuronal groups, in addition to the “optic” neuronal group itself. This confirms our previous hypothesis where we argued that the “visual projection” and “visual centrifugal” neuronal groups act as mediators between the “optic” and “central” neuronal groups. In the connectome motifs having “central” as their core group,“visual projection” appears mainly as an input group and “visual centrifugal” appears mainly as an output group. Interestingly, the analysis of connectome motifs having “visual centrifugal” as their core group (see Fig. 16) shows that there is a significant interaction between the “visual centrifugal” and “visual projection” neuronal groups, with an average number of connections between the two groups of about 120.

Now, let us consider the sixth connectome motif in Table 12; it is also the fifth connectome motif in Fig. 14). This motif has “central” as its core group, receives information only from the “visual projection” neuronal group, processes it in the “central” neuronal group, and sends the results only to the “descending” neuronal group; this motif has 122 occurrences, which means that there are 122 power neurons of the “central” core group having this functional structure. Interestingly, there is no frequent connectome motif that involves only one neuronal group; this reinforces the idea that power neurons are critical for managing interactions among different neuronal groups.

As we have already seen for the larva, some neuronal groups are almost completely absent from the connectome motifs of the adult. In particular, the “sensory” neuronal group has no power neurons and appears in only three infrequent motifs; the core group of these motifs is “central”; moreover, “sensory” always plays the role of input group in all of them. The “endocrine” neuronal group has no power neurons and is involved in only five infrequent motifs; in two of them the core group is “central”, while in the other three the core group is “descending”. In all these motifs, the “motor” group appears only as an output group with an average number of connections of about 2.

A final remark concerns the “ascending” neuronal group. Although it contains 50 power neurons, all connectome motifs with this core group are poorly represented (in particular, each of them has less than 10 occurrences). Moreover, whenever this neuronal group appears in connectome motifs with other core groups, it generally plays the role of input group (specifically, this happens in 80% of the cases).

## Conclusion

In this paper, we proposed an advancement in the study of neuronal connectomes based on the use of complex network analysis to define and characterize a particular type of neurons, which we call “power neurons”. These neurons are particularly interesting because of their structural connections within the connectome. We have focused on the connectome of *Drosophila melanogaster* in larval and adult stages. However, our approach to detecting and characterizing power neurons is general and can be extended to any connectome, including simpler ones, which are already known, and more complex ones, as they become available. Using complex network analysis to investigate the *Drosophila* connectome has provided us with a series of insights, which we have described in detail in the previous sections.

Specifically, our initial focus was the structural similarities and differences between the larval and adult connectomes. One insight we discovered is that the trend in the basic features of the *Drosophila* connectome when transitioning from the larval to adult stage (see Table 3) does not follow the typical trend of other complex networks (e.g., social, technological, and information networks), where density increases and diameter decreases as the network grows (Leskovec et al., 2005). In *Drosophila*, indeed, the transition from larval to adult stage is characterized by the following: *(i)* a significant increase in the number of neurons and connections (by two orders of magnitude for neurons and one order of magnitude for connections); *(ii)* a decrease in the average node degree and average clustering coefficient; *(iii)* a significant decrease in density (by two orders of magnitude); and *(iv)* essentially constant average path length and diameter. Finally, the maximum (strongly) connected component in both connectomes includes nearly all neurons, indicating that each neuron can potentially communicate with nearly any other neuron in the *Drosophila* connectome.

A second insight detected by our approach is that there is indeed a small set of power neurons in both the larval and adult *Drosophila* connectomes (see “Detecting Power Neurons” section). Power neurons advance the study of special neurons in the brain, such as *rich club neurons* (Lin et al., 2024). In fact, we have shown that power neurons are much fewer in number than *rich club neurons* (there are 60,848 *rich club neurons* and 5,473 power neurons in the adult *Drosophila* connectome). Despite their limited number, power neurons exhibit specific properties, including high centrality values and strong interconnections, leading them to form a backbone (see “Power Neurons Form a Backbone” section). This suggests that they play a critical role in coordinating the information flow within the connectome.

We also characterized power neurons in detail along different trajectories, namely: *(i)* the hemispheres and neuronal groups in which they are located, *(ii)* the subnetworks they induce, *(iii)* the connectome motifs in which they are involved, and so on.

In particular, as a third insight, we found that, although power neurons are evenly distributed between the two hemispheres, they are more prevalent in certain neuronal groups. This allows these groups to accumulate and aggregate information from different areas of the brain and the connectome (Figs. 8 and 9). Studying the direct connections of power neurons with other (power) neurons revealed a main set of neuronal groups connected by power neurons in both larval and adult *Drosophila* connectomes. This main set includes the “deep brain”, “learning/memory” and “innate” neuronal groups in the larval connectome (Fig. 10) and the “central”, “optic”, “visual projection” and “visual centrifugal” neuronal groups in the adult connectome (Fig. 11). These results highlight the distinct roles of different neuronal groups in processing signals within the connectome. We also found strong connections between neurons belonging to different hemispheres; these connections link both neurons of the same neuronal group and neurons of different neuronal groups.

We obtained a final insight by analyzing connectome motifs involving power neurons. Specifically, we found that power neurons are involved in several well-characterized motifs. These motifs reveal the patterns through which power neurons currently integrate different neuronal groups and interact with neurons of the same or other neuronal groups. Additionally, this analysis revealed that the larval connectome motifs are more homogeneous than the adult ones.

Summarizing, our findings not only contribute to the understanding of *Drosophila* biology, but also provide a framework for future investigations into neuronal connectomes of more complex organisms, when they become available.

Based on the exploratory analyses we have performed on larval and adult *Drosophila* connectomes, which we have only partially reported in this paper due to space limitations, it is possible to envisage several further developments of this research along different lines. In particular: *(i)* As indicated in this paper, we have found some evidence that there may be “special” power neurons within the set of power neurons; further analyses may lead to the identification of hierarchies of power neurons, perhaps characterized by different roles and tasks within the connectome. *(ii)* The study of connectome motifs can be extended to better understand the connections between power neurons and to determine whether there are connectome motifs more complex than the ones we found in this paper (e.g., think of connectome motifs that have a core triad of power neurons instead of a core power neuron). *(iii)* All previous studies show that there is a substantial symmetry between the two hemispheres, and the analyses shown in this paper also point in this direction. However, it would be interesting to investigate not only the symmetries, but also, and perhaps especially, the differences between the neurons of the two hemispheres, which might suggest the possible presence of some functions on which one hemisphere is more specialized than the other. *(iv)* We have coined the term neuronal group of a connectome and linked it to the concept of “annotation” in the larva and of “superclass” in the adult. It would be interesting to study the properties of the different neuronal groups in both the larva and the adult to try to understand their similarities and differences. *(v)* It would be interesting to investigate whether we can incorporate the complete *Drosophila* connectome and power neurons into computational models of the brain, as it was very recently done in Scheuermann et al. (2025). In particular, the properties of power neurons could be incorporated into such models and their effects on simulated behavior could be analyzed. *(vi)* It would be interesting to determine the specific functions of power neurons and how they influence the *Drosophila* behavior in different contexts. *(vii)* It would be challenging to explore whether power neurons are a common feature of different species, in which case interesting insights into the general principles of connectome organization could be derived. As data from the connectomes of increasingly complex organisms becomes available, our approach may serve as a valuable starting point for unraveling the relationships between brain structure and function across different species.

## Information Sharing Statement

All data and code used in this paper is available at https://github.com/FedericaParlapiano/PowerNeurons.git

## Supplementary Information

Below is the link to the electronic supplementary material.

Supplementary file 1 (pdf 380 KB)

## Data Availability

All data and code used in this paper is available at https://github.com/FedericaParlapiano/PowerNeurons.git

## Footnotes

1 See https://flywire.ai/.

2 The interested reader can find the complete list of statistically significant connectome motifs in additional material available at https://tinyurl.com/ywchnzrm
