# Supplementary material for: A Complex Network-Based Approach for Detecting and Characterizing Power Neurons in Drosophila
Source: Neuroinformatics. 2026 Feb 13;24(1):11. doi: 10.1007/s12021-026-09773-6 (PMC12904900; doi:10.1007/s12021-026-09773-6)
Supplement: Supplementary file 1 — (pdf 380 KB) [file 12021_2026_9773_MOESM1_ESM.pdf]

# A Complex Network-Based Approach for Detecting and Characterizing Power Neurons in Drosophila

Enrico Corradini[1], Federica Parlapiano[1], Giorgio Terracina[2*], Domenico Ursino[1]

[1] DII, Polytechnic University of Marche
[2] DEMACS, University of Calabria
[*] Contact Author
e.corradini@univpm.it; f.parlapiano@pm.univpm.it; giorgio.terracina@unical.it; d.ursino@univpm.it

## 1 Appendix 1: Statistically significant connectome motifs

### 1.1 Larva

| Input | Core group | Output | Count |
|---|---|---|---|
| brain outputs, deep brain, innate, learning/memory, pre-output | deep brain | brain outputs, deep brain, innate, learning/memory, pre-output | 26 |
| deep brain, innate, learning/memory, pre-output | learning/memory | brain outputs, deep brain, innate, learning/memory, pre-output | 13 |
| deep brain, innate, learning/memory, pre-output | deep brain | brain outputs, deep brain, innate, learning/memory, pre-output | 12 |
| brain outputs, deep brain, innate, learning/memory, pre-output | pre-output | brain outputs, deep brain, innate, learning/memory, pre-output | 8 |
| deep brain, innate, learning/memory, pre-output | learning/memory | deep brain, innate, learning/memory, pre-output | 7 |
| deep brain, innate, learning/memory, pre-output | innate | brain outputs, deep brain, innate, learning/memory, pre-output | 6 |
| ascending, brain outputs, deep brain, innate, learning/memory, pre-output | deep brain | brain outputs, deep brain, innate, learning/memory, pre-output | 5 |
| deep brain, innate, learning/memory, pre-output | innate | deep brain, innate, learning/memory, pre-output | 5 |
| brain outputs, deep brain, innate, learning/memory, pre-output | innate | brain outputs, deep brain, innate, learning/memory, pre-output | 5 |
| brain outputs, deep brain, innate, learning/memory, pre-output | learning/memory | brain outputs, deep brain, innate, learning/memory, pre-output | 4 |
| brain outputs, deep brain, innate, learning/memory, pre-output | innate | deep brain, innate, learning/memory, pre-output | 3 |
| brain outputs, deep brain, innate, learning/memory, pre-output | deep brain | deep brain, innate, learning/memory, pre-output | 3 |
| ascending, brain outputs, deep brain, innate, learning/memory, pre-output | deep brain | brain outputs, deep brain, innate, pre-output | 3 |

*Continued on next page*

| Input | Core group | Output | Count |
|---|---|---|---|
| ascending, brain outputs, deep brain, innate, learning/memory, pre-output, sensory | innate | brain outputs, deep brain, innate, learning/memory, pre-output | 3 |
| deep brain, innate, learning/memory, pre-output | deep brain | deep brain, innate, learning/memory, pre-output | 3 |
| brain outputs, deep brain, innate, learning/memory, pre-output, sensory | innate | brain outputs, deep brain, innate, learning/memory, pre-output | 2 |
| ascending, deep brain, innate, learning/memory, pre-output | innate | deep brain, innate, learning/memory, pre-output | 2 |
| ascending, deep brain, innate, learning/memory, pre-output | deep brain | brain outputs, deep brain, innate, learning/memory, pre-output | 2 |
| ascending, brain outputs, deep brain, innate, learning/memory, pre-output | deep brain | brain outputs, deep brain, learning/memory, pre-output | 2 |
| ascending, brain outputs, deep brain, innate, learning/memory, pre-output | pre-output | ascending, brain outputs, deep brain, innate, learning/memory, pre-output | 2 |
| brain outputs, deep brain, innate, learning/memory, pre-output | learning/memory | deep brain, innate, learning/memory | 2 |
| brain outputs, deep brain, innate, learning/memory, pre-output, sensory | learning/memory | brain outputs, deep brain, innate, learning/memory, pre-output | 2 |
| ascending, brain outputs, deep brain, innate, learning/memory, pre-output | brain outputs | ascending, brain outputs, deep brain, innate, pre-output | 1 |
| deep brain, innate, learning/memory, pre-output | deep brain | ascending, brain outputs, deep brain, innate, learning/memory, pre-output | 1 |
| brain outputs, deep brain, innate, learning/memory, pre-output, sensory | deep brain | brain outputs, deep brain, innate, learning/memory, pre-output | 1 |
| brain outputs, deep brain, innate, learning/memory, pre-output, sensory | deep brain | brain outputs, deep brain, innate, learning/memory, pre-output, sensory | 1 |
| deep brain, innate, learning/memory, pre-output | deep brain | brain outputs, deep brain, innate, pre-output | 1 |
| ascending, brain outputs, deep brain, innate, learning/memory, pre-output, sensory | deep brain | brain outputs, deep brain, innate, learning/memory, pre-output | 1 |
| ascending, deep brain, innate, learning/memory, pre-output | deep brain | brain outputs, deep brain, innate, pre-output | 1 |
| brain outputs, deep brain, innate, learning/memory, pre-output, sensory | brain outputs | ascending, brain outputs, deep brain, innate, learning/memory, pre-output, sensory | 1 |
| brain outputs, deep brain, innate, pre-output, sensory | brain outputs | ascending, brain outputs, deep brain, innate, learning/memory, pre-output, sensory | 1 |
| ascending, brain outputs, deep brain, innate, learning/memory, pre-output, sensory | brain outputs | brain outputs, deep brain, learning/memory, pre-output | 1 |
| ascending, brain outputs, deep brain, innate, pre-output | brain outputs | brain outputs, deep brain, innate, learning/memory, pre-output | 1 |
| ascending, brain outputs, deep brain, innate, learning/memory, pre-output, sensory | brain outputs | brain outputs, deep brain, innate, learning/memory, pre-output | 1 |
| brain outputs, deep brain, innate, learning/memory, pre-output, sensory | innate | ascending, brain outputs, deep brain, innate, learning/memory, pre-output | 1 |
| ascending, brain outputs, deep brain, innate, learning/memory, pre-output | innate | brain outputs, deep brain, innate, learning/memory, pre-output | 1 |
| ascending, brain outputs, deep brain, innate, learning/memory, pre-output | innate | ascending, brain outputs, deep brain, innate, learning/memory, pre-output | 1 |
| ascending, deep brain, innate, learning/memory, pre-output | innate | brain outputs, deep brain, innate, learning/memory, pre-output | 1 |
| brain outputs, deep brain, innate, pre-output, sensory | innate | brain outputs, deep brain, innate, learning/memory, pre-output | 1 |
| deep brain, innate, learning/memory, pre-output | innate | ascending, brain outputs, deep brain, innate, learning/memory, pre-output | 1 |
| brain outputs, deep brain, innate, learning/memory, pre-output | innate | ascending, brain outputs, deep brain, innate, learning/memory, pre-output | 1 |

*Continued on next page*

| Input | Core group | Output | Count |
| --- | --- | --- | --- |
| brain outputs, deep brain, innate, learning/memory, pre-output | innate | brain outputs, deep brain, innate, learning/memory, pre-output, sensory | 1 |
| deep brain, innate, learning/memory, pre-output | innate | innate, learning/memory | 1 |
| brain outputs, deep brain, innate, learning/memory, pre-output | learning/memory | deep brain, innate, learning/memory, pre-output | 1 |
| brain outputs, deep brain, innate, learning/memory, pre-output | learning/memory | innate, learning/memory | 1 |
| innate, learning/memory | learning/memory | deep brain, innate, learning/memory, pre-output | 1 |
| deep brain, innate, learning/memory, pre-output | learning/memory | brain outputs, deep brain, innate, learning/memory | 1 |
| brain outputs, deep brain, innate, learning/memory | learning/memory | brain outputs, deep brain, innate, learning/memory, pre-output | 1 |
| deep brain, innate, learning/memory, pre-output | learning/memory | learning/memory | 1 |
| ascending, brain outputs, deep brain, innate, learning/memory, pre-output | learning/memory | deep brain, innate, learning/memory | 1 |
| ascending, brain outputs, deep brain, innate, learning/memory, pre-output | learning/memory | brain outputs, deep brain, innate, learning/memory | 1 |
| deep brain, innate, learning/memory, pre-output, sensory | learning/memory | deep brain, innate, learning/memory, pre-output | 1 |
| brain outputs, deep brain, innate, learning/memory, pre-output | pre-output | deep brain, innate, learning/memory, pre-output | 1 |
| brain outputs, deep brain, innate, pre-output | pre-output | brain outputs, innate, pre-output | 1 |
| brain outputs, deep brain, innate, learning/memory, pre-output, sensory | pre-output | brain outputs, deep brain, innate, pre-output | 1 |

## 1.2 Adult

| Input | Core group | Output | Count |
| --- | --- | --- | --- |
| ascending, central, visual projection | central | central, descending | 202 |
| central, optic, visual centrifugal, visual projection | visual projection | central, descending, optic, visual centrifugal, visual projection | 197 |
| ascending, central, visual projection | central | central, descending, visual centrifugal | 188 |
| ascending, central, descending, visual projection | central | central, descending, visual centrifugal | 158 |
| central, visual projection | central | central, descending, visual centrifugal | 125 |
| optic, visual centrifugal, visual projection | optic | optic, visual projection | 122 |
| central, visual projection | central | central, descending | 122 |
| ascending, central, descending, visual projection | central | central, descending | 117 |
| central, visual projection | central | central | 106 |
| central, optic, visual centrifugal, visual projection | visual projection | central, descending, optic, visual projection | 103 |
| central, optic, visual centrifugal, visual projection | visual projection | central, visual centrifugal | 102 |
| ascending, central, visual projection | central | central | 89 |
| central, optic, visual centrifugal, visual projection | visual projection | central, visual centrifugal, visual projection | 85 |
| ascending, central, descending, visual centrifugal, visual projection | central | central, descending, visual centrifugal, visual projection | 81 |
| ascending, central, visual projection | central | central, descending, visual centrifugal, visual projection | 80 |

*Continued on next page*

| Input | Core group | Output | Count |
|---|---|---|---|
| central, optic, visual centrifugal, visual projection | visual projection | central, descending, visual centrifugal, visual projection | 80 |
| optic, visual centrifugal, visual projection | optic | optic, visual centrifugal, visual projection | 79 |
| ascending, central, descending, visual projection | central | central, descending, visual centrifugal, visual projection | 70 |
| central, optic, visual centrifugal, visual projection | visual projection | central, optic, visual centrifugal, visual projection | 69 |
| ascending, central, descending, visual centrifugal, visual projection | central | central, descending, visual centrifugal | 67 |
| central, visual projection | central | central, visual projection | 61 |
| central, visual projection | central | central, visual centrifugal | 58 |
| ascending, central, visual centrifugal, visual projection | central | central, descending, visual centrifugal | 58 |
| ascending, central, descending | central | central, descending, visual centrifugal | 51 |
| ascending, central, visual projection | central | central, visual projection | 50 |
| central, visual projection | central | central, descending, visual centrifugal, visual projection | 48 |
| ascending, central, visual centrifugal, visual projection | central | central, descending, visual centrifugal, visual projection | 45 |
| ascending, central, descending | central | central, descending | 45 |
| central | central | central, descending, visual centrifugal | 44 |
| central, optic, visual centrifugal, visual projection | visual projection | central, optic, visual projection | 43 |
| ascending, central, visual projection | central | central, descending, visual projection | 43 |
| ascending, central, descending, visual projection | central | ascending, central, descending, visual centrifugal | 43 |
| central | central | central, descending | 38 |
| central, visual projection | central | central, descending, visual projection | 37 |
| ascending, central | central | central, descending | 36 |
| ascending, central, descending, visual projection | central | ascending, central, descending | 36 |
| ascending, central, descending | central | ascending, central, descending | 33 |
| ascending, central, descending, visual centrifugal, visual projection | central | ascending, central, descending, visual centrifugal, visual projection | 32 |
| ascending, central, visual projection | central | central, visual centrifugal | 31 |
| ascending, central, optic, visual centrifugal, visual projection | visual projection | central, descending, optic, visual centrifugal, visual projection | 31 |
| ascending, central | central | central, descending, visual centrifugal | 31 |
| central, descending, visual projection | central | central, descending, visual centrifugal | 30 |
| ascending, central, descending, visual projection | central | ascending, central, descending, visual centrifugal, visual projection | 29 |
| ascending, central, visual projection | central | central, visual centrifugal, visual projection | 25 |
| ascending, central, descending | central | central | 25 |
| central, visual projection | central | central, visual centrifugal, visual projection | 24 |
| central, visual centrifugal, visual projection | central | central, descending, visual centrifugal, visual projection | 23 |
| ascending, central, descending, optic, visual centrifugal, visual projection | visual centrifugal | central, optic, visual centrifugal, visual projection | 23 |
| central, visual centrifugal, visual projection | central | central, descending, visual centrifugal | 23 |
| ascending, central, visual centrifugal, visual projection | central | central, visual centrifugal, visual projection | 22 |
| ascending, central, descending, visual projection | descending | central, descending, motor | 21 |
| ascending, central, descending, visual projection | descending | central, descending | 21 |
| central | central | central | 21 |

*Continued on next page*

| Input | Core group | Output | Count |
| --- | --- | --- | --- |
| ascending, central, descending, visual projection | central | ascending, central, descending, visual projection | 21 |
| ascending, central, descending, visual projection | descending | ascending, central, descending | 20 |
| ascending, central, descending, visual projection | descending | central, descending, motor, visual centrifugal | 20 |
| ascending, central | central | central | 20 |
| ascending, central, descending, visual projection | central | central, descending, motor, visual centrifugal | 20 |
| ascending, central, descending, sensory | central | ascending, central, descending | 19 |
| ascending, central, visual centrifugal, visual projection | central | central, visual centrifugal | 19 |
| ascending, central, descending, visual projection | central | central, descending, visual projection | 19 |
| ascending, central, visual projection | central | ascending, central, descending, visual projection | 19 |
| ascending, central, descending | central | ascending, central, descending, visual centrifugal | 19 |
| ascending, central, descending, optic, visual centrifugal, visual projection | visual centrifugal | central, descending, optic, visual centrifugal, visual projection | 19 |
| central, optic, visual centrifugal, visual projection | visual projection | central, descending, visual projection | 19 |
| central | central | central, descending, visual centrifugal, visual projection | 18 |
| ascending, central, visual centrifugal, visual projection | central | central, descending | 18 |
| central, descending, visual projection | central | central, descending | 18 |
| ascending, central, descending, visual projection | descending | central, descending, visual centrifugal | 17 |
| central, visual centrifugal, visual projection | central | central, descending | 17 |
| optic, visual centrifugal, visual projection | optic | descending, optic, visual projection | 17 |
| ascending, central, descending, sensory, visual projection | central | central, descending, visual centrifugal | 16 |
| ascending, central, descending, visual centrifugal, visual projection | central | central, descending, motor, visual centrifugal | 16 |
| central, optic, visual centrifugal | visual projection | central, descending, optic, visual projection | 16 |
| ascending, central, descending, optic, visual centrifugal, visual projection | visual centrifugal | optic, visual centrifugal, visual projection | 15 |
| ascending, central, visual projection | central | ascending, central, descending, visual centrifugal, visual projection | 15 |
| ascending, central, descending, visual centrifugal, visual projection | central | central, descending | 15 |
| central, optic, visual centrifugal | visual projection | central, optic, visual centrifugal, visual projection | 15 |
| ascending, central, descending, visual centrifugal, visual projection | descending | central, descending | 14 |
| ascending, central, visual projection | central | ascending, central, descending, visual centrifugal | 14 |
| ascending, central, descending, visual projection | central | central | 14 |
| ascending, central, descending, visual centrifugal, visual projection | descending | central, descending, motor | 14 |
| ascending, central, visual projection | central | ascending, central, descending | 14 |
| ascending, central, descending, visual centrifugal, visual projection | descending | central, descending, visual centrifugal | 13 |
| ascending, central, descending, sensory | central | central, descending, visual centrifugal | 13 |

*Continued on next page*

| Input | Core group | Output | Count |
| --- | --- | --- | --- |
| central, descending | central | central, descending | 12 |
| ascending, central, descending | central | central, descending, visual centrifugal, visual projection | 12 |
| central, descending, visual centrifugal, visual projection | central | central, descending, visual centrifugal | 12 |
| ascending, central, descending, visual projection | descending | ascending, central, descending, visual centrifugal | 12 |
| central, descending | central | central, descending, visual centrifugal | 12 |
| central, descending, visual projection | central | central, descending, visual centrifugal, visual projection | 12 |
| central | central | central, visual centrifugal | 11 |
| ascending, central, visual projection | central | ascending, central, visual projection | 11 |
| central, optic, visual centrifugal, visual projection | visual projection | central, visual projection | 11 |
| ascending, central, optic, visual centrifugal, visual projection | visual projection | central, descending, optic, visual projection | 11 |

# 2 Appendix 2: Graphical representation of connectome motifs

## 2.1 Larva

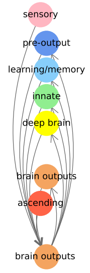


Motif count: 1

**Incoming arcs**:
('ascending', 'brain-outputs'): 2.0,
('brain-outputs', 'brain-outputs'): 12.0,
('deep-brain', 'brain-outputs'): 22.0,
('innate', 'brain-outputs'): 14.0,
('learning/memory', 'brain-outputs'): 2.0,
('pre-output', 'brain-outputs'): 60.0,
('sensory', 'brain-outputs'): 1.0

**Outgoing arcs**:
('brain-outputs', 'brain-outputs'): 16.0,
('brain-outputs', 'deep-brain'): 5.0,
('brain-outputs', 'innate'): 6.0,
('brain-outputs', 'learning/memory'): 1.0,
('brain-outputs', 'pre-output'): 8.0

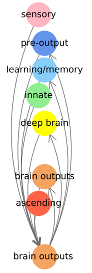


Motif count: 1

**Incoming arcs**:
('ascending', 'brain-outputs'): 1.0,
('brain-outputs', 'brain-outputs'): 10.0,
('deep-brain', 'brain-outputs'): 20.0,
('innate', 'brain-outputs'): 10.0,
('learning/memory', 'brain-outputs'): 2.0,
('pre-output', 'brain-outputs'): 46.0,
('sensory', 'brain-outputs'): 2.0

**Outgoing arcs**:
('brain-outputs', 'brain-outputs'): 17.0,
('brain-outputs', 'deep-brain'): 2.0,
('brain-outputs', 'learning/memory'): 1.0,
('brain-outputs', 'pre-output'): 4.0

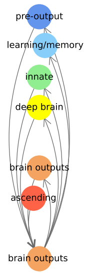


Motif count: 1

**Incoming arcs**:
('ascending', 'brain-outputs'): 1.0,
('brain-outputs', 'brain-outputs'): 13.0,
('deep-brain', 'brain-outputs'): 18.0,
('innate', 'brain-outputs'): 11.0,
('pre-output', 'brain-outputs'): 52.0

**Outgoing arcs**:
('brain-outputs', 'brain-outputs'): 13.0,
('brain-outputs', 'deep-brain'): 2.0,
('brain-outputs', 'innate'): 1.0,
('brain-outputs', 'learning/memory'): 2.0,
('brain-outputs', 'pre-output'): 5.0

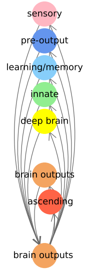


Motif count: 1

**Incoming arcs**:
('brain-outputs', 'brain-outputs'): 25.0,
('deep-brain', 'brain-outputs'): 14.0,
('innate', 'brain-outputs'): 37.0,
('learning/memory', 'brain-outputs'): 2.0,
('pre-output', 'brain-outputs'): 28.0,
('sensory', 'brain-outputs'): 7.0

**Outgoing arcs**:
('brain-outputs', 'ascending'): 1.0,
('brain-outputs', 'brain-outputs'): 37.0,
('brain-outputs', 'deep-brain'): 3.0,
('brain-outputs', 'innate'): 7.0,
('brain-outputs', 'learning/memory'): 1.0,
('brain-outputs', 'pre-output'): 3.0,
('brain-outputs', 'sensory'): 2.0

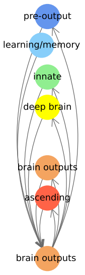


Motif count: 1

**Incoming arcs**:
('ascending', 'brain-outputs'): 1.0,
('brain-outputs', 'brain-outputs'): 16.0,
('deep-brain', 'brain-outputs'): 20.0,
('innate', 'brain-outputs'): 22.0,
('learning/memory', 'brain-outputs'): 1.0,
('pre-output', 'brain-outputs'): 43.0

**Outgoing arcs**:
('brain-outputs', 'ascending'): 1.0,
('brain-outputs', 'brain-outputs'): 13.0,
('brain-outputs', 'deep-brain'): 4.0,
('brain-outputs', 'innate'): 6.0,
('brain-outputs', 'pre-output'): 5.0

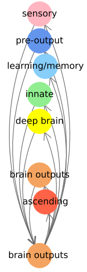


Motif count: 1

**Incoming arcs**:
('brain-outputs', 'brain-outputs'): 24.0,
('deep-brain', 'brain-outputs'): 13.0,
('innate', 'brain-outputs'): 27.0,
('pre-output', 'brain-outputs'): 30.0,
('sensory', 'brain-outputs'): 1.0

**Outgoing arcs**:
('brain-outputs', 'ascending'): 1.0,
('brain-outputs', 'brain-outputs'): 31.0,
('brain-outputs', 'deep-brain'): 2.0,
('brain-outputs', 'innate'): 7.0,
('brain-outputs', 'learning/memory'): 1.0,
('brain-outputs', 'pre-output'): 3.0,
('brain-outputs', 'sensory'): 3.0

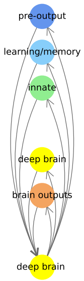


Motif count: 26

**Incoming arcs**:
('brain-outputs', 'deep-brain'): 1.73,
('deep-brain', 'deep-brain'): 29.65,
('innate', 'deep-brain'): 29.73,
('learning/memory', 'deep-brain'): 16.35,
('pre-output', 'deep-brain'): 18.58

**Outgoing arcs:**
('deep-brain', 'brain-outputs'): 6.12,
('deep-brain', 'deep-brain'): 25.92,
('deep-brain', 'innate'): 12.23,
('deep-brain', 'learning/memory'): 8.23,
('deep-brain', 'pre-output'): 15.23

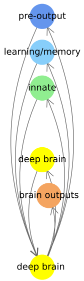


Motif count: 12

**Incoming arcs**:
('deep-brain', 'deep-brain'): 27.92,
('innate', 'deep-brain'): 33.67,
('learning/memory', 'deep-brain'): 13.83,
('pre-output', 'deep-brain'): 10.5

**Outgoing arcs:**
('deep-brain', 'brain-outputs'): 6.17,
('deep-brain', 'deep-brain'): 23.17,
('deep-brain', 'innate'): 9.33,
('deep-brain', 'learning/memory'): 5.67,
('deep-brain', 'pre-output'): 16.75

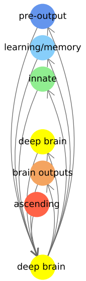


Motif count: 5

**Incoming arcs**:
('ascending', 'deep-brain'): 1.2,
('brain-outputs', 'deep-brain'): 6.2,
('deep-brain', 'deep-brain'): 17.8,
('innate', 'deep-brain'): 35.2,
('learning/memory', 'deep-brain'): 3.6,
('pre-output', 'deep-brain'): 15.0

**Outgoing arcs:**
('deep-brain', 'brain-outputs'): 8.6,
('deep-brain', 'deep-brain'): 19.4,
('deep-brain', 'innate'): 9.0,
('deep-brain', 'learning/memory'): 5.4,
('deep-brain', 'pre-output'): 17.6

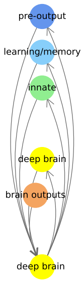


Motif count: 3

**Incoming arcs**:
('brain-outputs', 'deep-brain'): 1.0,
('deep-brain', 'deep-brain'): 17.67,
('innate', 'deep-brain'): 43.67,
('learning/memory', 'deep-brain'): 11.67,
('pre-output', 'deep-brain'): 12.33

**Outgoing arcs:**
('deep-brain', 'deep-brain'): 32.0,
('deep-brain', 'innate'): 6.33,
('deep-brain', 'learning/memory'): 12.67,
('deep-brain', 'pre-output'): 4.67

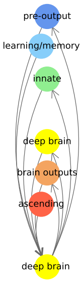


Motif count: 3

**Incoming arcs**:
('ascending', 'deep-brain'): 1.0,
('brain-outputs', 'deep-brain'): 7.33,
('deep-brain', 'deep-brain'): 22.67,
('innate', 'deep-brain'): 17.67,
('learning/memory', 'deep-brain'): 10.0,
('pre-output', 'deep-brain'): 33.0

**Outgoing arcs:**
('deep-brain', 'brain-outputs'): 11.33,
('deep-brain', 'deep-brain'): 3.33,
('deep-brain', 'innate'): 3.33,
('deep-brain', 'pre-output'): 8.0

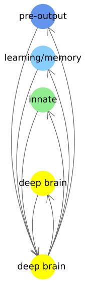


Motif count: 3

**Incoming arcs**:
('deep-brain', 'deep-brain'): 32.33,
('innate', 'deep-brain'): 33.33,
('learning/memory', 'deep-brain'): 14.33,
('pre-output', 'deep-brain'): 7.33

**Outgoing arcs:**
('deep-brain', 'deep-brain'): 38.33,
('deep-brain', 'innate'): 23.33,
('deep-brain', 'learning/memory'): 15.33,
('deep-brain', 'pre-output'): 9.67

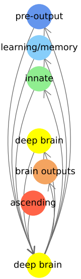


Motif count: 2

**Incoming arcs**:
('ascending', 'deep-brain'): 1.0,
('deep-brain', 'deep-brain'): 24.0,
('innate', 'deep-brain'): 37.5,
('learning/memory', 'deep-brain'): 9.5,
('pre-output', 'deep-brain'): 12.0

**Outgoing arcs**:
('deep-brain', 'brain-outputs'): 4.5,
('deep-brain', 'deep-brain'): 20.0,
('deep-brain', 'innate'): 17.0,
('deep-brain', 'learning/memory'): 4.0,
('deep-brain', 'pre-output'): 9.0

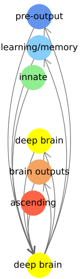


Motif count: 2

**Incoming arcs**:
('ascending', 'deep-brain'): 1.0,
('brain-outputs', 'deep-brain'): 19.0,
('deep-brain', 'deep-brain'): 18.0,
('innate', 'deep-brain'): 17.0,
('learning/memory', 'deep-brain'): 5.0,
('pre-output', 'deep-brain'): 20.5

**Outgoing arcs**:
('deep-brain', 'brain-outputs'): 13.5,
('deep-brain', 'deep-brain'): 11.5,
('deep-brain', 'learning/memory'): 2.0,
('deep-brain', 'pre-output'): 11.0

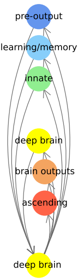


Motif count: 1

**Incoming arcs**:
('deep-brain', 'deep-brain'): 8.0,
('innate', 'deep-brain'): 26.0,
('learning/memory', 'deep-brain'): 8.0,
('pre-output', 'deep-brain'): 10.0

**Outgoing arcs**:
('deep-brain', 'ascending'): 1.0,
('deep-brain', 'brain-outputs'): 2.0,
('deep-brain', 'deep-brain'): 12.0,
('deep-brain', 'innate'): 40.0,
('deep-brain', 'learning/memory'): 4.0,
('deep-brain', 'pre-output'): 9.0

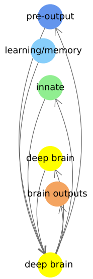


Motif count: 1

**Incoming arcs**:
('deep-brain', 'deep-brain'): 18.0,
('innate', 'deep-brain'): 22.0,
('learning/memory', 'deep-brain'): 12.0,
('pre-output', 'deep-brain'): 23.0

**Outgoing arcs**:
('deep-brain', 'brain-outputs'): 5.0,
('deep-brain', 'deep-brain'): 5.0,
('deep-brain', 'innate'): 7.0,
('deep-brain', 'pre-output'): 19.0

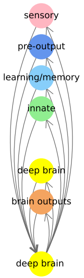


Motif count: 1

**Incoming arcs**:
('brain-outputs', 'deep-brain'): 6.0,
('deep-brain', 'deep-brain'): 6.0,
('innate', 'deep-brain'): 26.0,
('learning/memory', 'deep-brain'): 6.0,
('pre-output', 'deep-brain'): 11.0,
('sensory', 'deep-brain'): 5.0

**Outgoing arcs**:
('deep-brain', 'brain-outputs'): 29.0,
('deep-brain', 'deep-brain'): 3.0,
('deep-brain', 'innate'): 19.0,
('deep-brain', 'learning/memory'): 1.0,
('deep-brain', 'pre-output'): 7.0,
('deep-brain', 'sensory'): 1.0

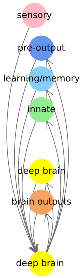


Motif count: 1

**Incoming arcs**:
('brain-outputs', 'deep-brain'): 4.0,
('deep-brain', 'deep-brain'): 7.0,
('innate', 'deep-brain'): 33.0,
('learning/memory', 'deep-brain'): 9.0,
('pre-output', 'deep-brain'): 9.0,
('sensory', 'deep-brain'): 3.0

**Outgoing arcs**:
('deep-brain', 'brain-outputs'): 27.0,
('deep-brain', 'deep-brain'): 4.0,
('deep-brain', 'innate'): 13.0,
('deep-brain', 'learning/memory'): 2.0,
('deep-brain', 'pre-output'): 13.0

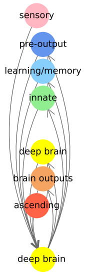


Motif count: 1

**Incoming arcs**:
('ascending', 'deep-brain'): 1.0,
('brain-outputs', 'deep-brain'): 9.0,
('deep-brain', 'deep-brain'): 20.0,
('innate', 'deep-brain'): 7.0,
('learning/memory', 'deep-brain'): 3.0,
('pre-output', 'deep-brain'): 20.0,
('sensory', 'deep-brain'): 1.0

**Outgoing arcs**:
('deep-brain', 'brain-outputs'): 10.0,
('deep-brain', 'deep-brain'): 17.0,
('deep-brain', 'innate'): 2.0,
('deep-brain', 'learning/memory'): 3.0,
('deep-brain', 'pre-output'): 19.0

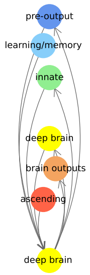


Motif count: 1

**Incoming arcs**:
('ascending', 'deep-brain'): 1.0,
('deep-brain', 'deep-brain'): 17.0,
('innate', 'deep-brain'): 26.0,
('learning/memory', 'deep-brain'): 8.0,
('pre-output', 'deep-brain'): 13.0

**Outgoing arcs**:
('deep-brain', 'brain-outputs'): 2.0,
('deep-brain', 'deep-brain'): 5.0,
('deep-brain', 'innate'): 17.0,
('deep-brain', 'pre-output'): 6.0

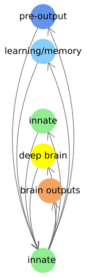


Motif count: 6

**Incoming arcs**:
('deep-brain', 'innate'): 15.83,
('innate', 'innate'): 33.33,
('learning/memory', 'innate'): 6.83,
('pre-output', 'innate'): 18.17

**Outgoing arcs**:
('innate', 'brain-outputs'): 8.67,
('innate', 'deep-brain'): 14.0,
('innate', 'innate'): 17.83,
('innate', 'learning/memory'): 3.83,
('innate', 'pre-output'): 15.67

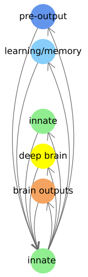


Motif count: 5

**Incoming arcs**:
('brain-outputs', 'innate'): 2.0,
('deep-brain', 'innate'): 8.4,
('innate', 'innate'): 27.6,
('learning/memory', 'innate'): 5.6,
('pre-output', 'innate'): 18.2

**Outgoing arcs**:
('innate', 'brain-outputs'): 5.0,
('innate', 'deep-brain'): 18.2,
('innate', 'innate'): 20.8,
('innate', 'learning/memory'): 8.0,
('innate', 'pre-output'): 10.6

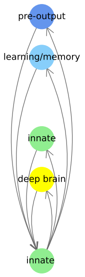


Motif count: 5

**Incoming arcs**:
('deep-brain', 'innate'): 16.8,
('innate', 'innate'): 45.0,
('learning/memory', 'innate'): 8.2,
('pre-output', 'innate'): 13.8

**Outgoing arcs**:
('innate', 'deep-brain'): 27.4,
('innate', 'innate'): 22.4,
('innate', 'learning/memory'): 9.2,
('innate', 'pre-output'): 4.0

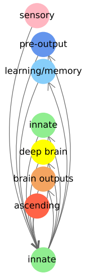


Motif count: 3

**Incoming arcs**:
('ascending', 'innate'): 1.0,
('brain-outputs', 'innate'): 8.67,
('deep-brain', 'innate'): 23.33,
('innate', 'innate'): 23.67,
('learning/memory', 'innate'): 2.67,
('pre-output', 'innate'): 37.67,
('sensory', 'innate'): 3.33

**Outgoing arcs**:
('innate', 'brain-outputs'): 6.33,
('innate', 'deep-brain'): 23.67,
('innate', 'innate'): 9.0,
('innate', 'learning/memory'): 8.33,
('innate', 'pre-output'): 7.33

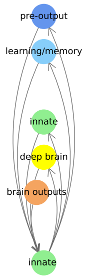


Motif count: 3

**Incoming arcs**:
('brain-outputs', 'innate'): 1.33,
('deep-brain', 'innate'): 7.67,
('innate', 'innate'): 62.0,
('learning/memory', 'innate'): 4.0,
('pre-output', 'innate'): 10.33

**Outgoing arcs**:
('innate', 'deep-brain'): 20.33,
('innate', 'innate'): 25.67,
('innate', 'learning/memory'): 4.67,
('innate', 'pre-output'): 5.67

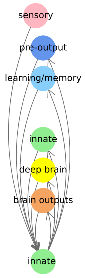


Motif count: 2

**Incoming arcs**:
('brain-outputs', 'innate'): 7.5,
('deep-brain', 'innate'): 11.5,
('innate', 'innate'): 16.5,
('learning/memory', 'innate'): 5.0,
('pre-output', 'innate'): 23.0,
('sensory', 'innate'): 8.0

**Outgoing arcs**:
('innate', 'brain-outputs'): 5.5,
('innate', 'deep-brain'): 19.5,
('innate', 'innate'): 12.0,
('innate', 'learning/memory'): 8.0,
('innate', 'pre-output'): 5.5

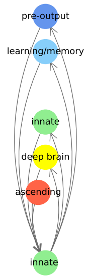


Motif count: 2

**Incoming arcs**:
('ascending', 'innate'): 1.5,
('deep-brain', 'innate'): 12.5,
('innate', 'innate'): 40.0,
('learning/memory', 'innate'): 3.0,
('pre-output', 'innate'): 21.0

**Outgoing arcs**:
('innate', 'deep-brain'): 20.5,
('innate', 'innate'): 16.0,
('innate', 'learning/memory'): 7.5,
('innate', 'pre-output'): 7.5

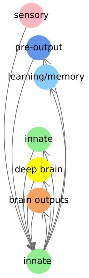


Motif count: 1

**Incoming arcs**:
('brain-outputs', 'innate'): 10.0,
('deep-brain', 'innate'): 30.0,
('innate', 'innate'): 23.0,
('pre-output', 'innate'): 24.0,
('sensory', 'innate'): 3.0

**Outgoing arcs**:
('innate', 'brain-outputs'): 8.0,
('innate', 'deep-brain'): 34.0,
('innate', 'innate'): 15.0,
('innate', 'learning/memory'): 11.0,
('innate', 'pre-output'): 11.0

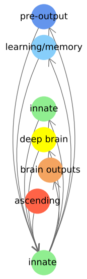


Motif count: 1

**Incoming arcs**:
('ascending', 'innate'): 1.0,
('deep-brain', 'innate'): 24.0,
('innate', 'innate'): 31.0,
('learning/memory', 'innate'): 1.0,
('pre-output', 'innate'): 25.0

**Outgoing arcs**:
('innate', 'brain-outputs'): 2.0,
('innate', 'deep-brain'): 29.0,
('innate', 'innate'): 6.0,
('innate', 'learning/memory'): 10.0,
('innate', 'pre-output'): 10.0

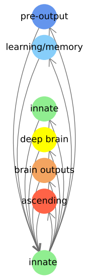


Motif count: 1

**Incoming arcs**:
('ascending', 'innate'): 1.0,
('brain-outputs', 'innate'): 1.0,
('deep-brain', 'innate'): 5.0,
('innate', 'innate'): 39.0,
('learning/memory', 'innate'): 8.0,
('pre-output', 'innate'): 3.0

**Outgoing arcs**:
('innate', 'ascending'): 1.0,
('innate', 'brain-outputs'): 1.0,
('innate', 'deep-brain'): 16.0,
('innate', 'innate'): 51.0,
('innate', 'learning/memory'): 2.0,
('innate', 'pre-output'): 15.0

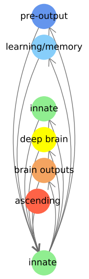


Motif count: 1

**Incoming arcs**:
('ascending', 'innate'): 1.0,
('brain-outputs', 'innate'): 2.0,
('deep-brain', 'innate'): 18.0,
('innate', 'innate'): 33.0,
('learning/memory', 'innate'): 2.0,
('pre-output', 'innate'): 16.0

**Outgoing arcs**:
('innate', 'brain-outputs'): 4.0,
('innate', 'deep-brain'): 8.0,
('innate', 'innate'): 17.0,
('innate', 'learning/memory'): 2.0,
('innate', 'pre-output'): 13.0

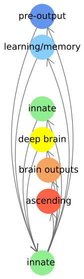


Motif count: 1

**Incoming arcs**:
('deep-brain', 'innate'): 7.0,
('innate', 'innate'): 49.0,
('learning/memory', 'innate'): 6.0,
('pre-output', 'innate'): 6.0

**Outgoing arcs**:
('innate', 'ascending'): 1.0,
('innate', 'brain-outputs'): 1.0,
('innate', 'deep-brain'): 18.0,
('innate', 'innate'): 26.0,
('innate', 'learning/memory'): 2.0,
('innate', 'pre-output'): 15.0

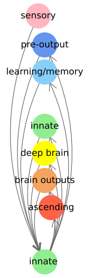


Motif count: 1

**Incoming arcs**:
('brain-outputs', 'innate'): 4.0,
('deep-brain', 'innate'): 1.0,
('innate', 'innate'): 47.0,
('learning/memory', 'innate'): 10.0,
('pre-output', 'innate'): 4.0,
('sensory', 'innate'): 4.0

**Outgoing arcs**:
('innate', 'ascending'): 1.0,
('innate', 'brain-outputs'): 1.0,
('innate', 'deep-brain'): 3.0,
('innate', 'innate'): 62.0,
('innate', 'learning/memory'): 3.0,
('innate', 'pre-output'): 10.0

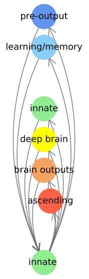


Motif count: 1

**Incoming arcs**:
('brain-outputs', 'innate'): 1.0,
('deep-brain', 'innate'): 4.0,
('innate', 'innate'): 41.0,
('learning/memory', 'innate'): 11.0,
('pre-output', 'innate'): 9.0

**Outgoing arcs**:
('innate', 'ascending'): 1.0,
('innate', 'brain-outputs'): 1.0,
('innate', 'deep-brain'): 12.0,
('innate', 'innate'): 45.0,
('innate', 'learning/memory'): 8.0,
('innate', 'pre-output'): 11.0

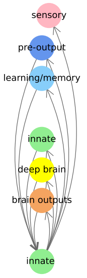


Motif count: 1

**Incoming arcs**:
('brain-outputs', 'innate'): 1.0,
('deep-brain', 'innate'): 15.0,
('innate', 'innate'): 75.0,
('learning/memory', 'innate'): 5.0,
('pre-output', 'innate'): 17.0

**Outgoing arcs**:
('innate', 'brain-outputs'): 35.0,
('innate', 'deep-brain'): 6.0,
('innate', 'innate'): 22.0,
('innate', 'learning/memory'): 1.0,
('innate', 'pre-output'): 6.0,
('innate', 'sensory'): 1.0

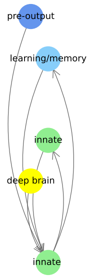


Motif count: 1

**Incoming arcs**:
('deep-brain', 'innate'): 7.0,
('innate', 'innate'): 25.0,
('learning/memory', 'innate'): 90.0,
('pre-output', 'innate'): 1.0

**Outgoing arcs**:
('innate', 'innate'): 20.0,
('innate', 'learning/memory'): 76.0

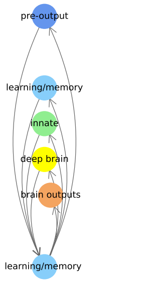


Motif count: 13

**Incoming arcs**:
('deep-brain', 'learning/memory'): 13.85,
('innate', 'learning/memory'): 14.08,
('learning/memory', 'learning/memory'): 74.77,
('pre-output', 'learning/memory'): 5.62

**Outgoing arcs:**
('learning/memory', 'brain-outputs'): 4.15,
('learning/memory', 'deep-brain'): 29.92,
('learning/memory', 'innate'): 12.46,
('learning/memory', 'learning/memory'): 14.31,
('learning/memory', 'pre-output'): 16.62

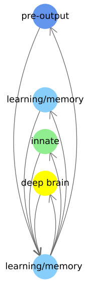


Motif count: 7

**Incoming arcs**:
('deep-brain', 'learning/memory'): 15.43,
('innate', 'learning/memory'): 21.71,
('learning/memory', 'learning/memory'): 70.43,
('pre-output', 'learning/memory'): 6.0

**Outgoing arcs:**
('learning/memory', 'deep-brain'): 11.0,
('learning/memory', 'innate'): 11.43,
('learning/memory', 'learning/memory'): 45.14,
('learning/memory', 'pre-output'): 3.29

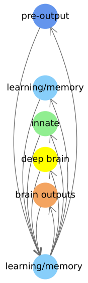


Motif count: 4

**Incoming arcs**:
('brain-outputs', 'learning/memory'): 1.5,
('deep-brain', 'learning/memory'): 16.25,
('innate', 'learning/memory'): 27.5,
('learning/memory', 'learning/memory'): 53.75,
('pre-output', 'learning/memory'): 14.25

**Outgoing arcs:**
('learning/memory', 'brain-outputs'): 3.5,
('learning/memory', 'deep-brain'): 11.75,
('learning/memory', 'innate'): 7.5,
('learning/memory', 'learning/memory'): 43.75,
('learning/memory', 'pre-output'): 10.5

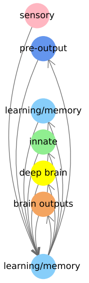


Motif count: 2

**Incoming arcs**:
('brain-outputs', 'learning/memory'): 11.0,
('deep-brain', 'learning/memory'): 12.0,
('innate', 'learning/memory'): 15.5,
('learning/memory', 'learning/memory'): 21.5,
('pre-output', 'learning/memory'): 2.0,
('sensory', 'learning/memory'): 1.0

**Outgoing arcs:**
('learning/memory', 'brain-outputs'): 4.0,
('learning/memory', 'deep-brain'): 18.0,
('learning/memory', 'innate'): 9.5,
('learning/memory', 'learning/memory'): 20.0,
('learning/memory', 'pre-output'): 4.0

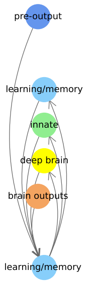


Motif count: 2

**Incoming arcs**:
('brain-outputs', 'learning/memory'): 1.5,
('deep-brain', 'learning/memory'): 36.5,
('innate', 'learning/memory'): 20.0,
('learning/memory', 'learning/memory'): 115.0,
('pre-output', 'learning/memory'): 11.0

**Outgoing arcs:**
('learning/memory', 'deep-brain'): 2.0,
('learning/memory', 'innate'): 1.5,
('learning/memory', 'learning/memory'): 111.0

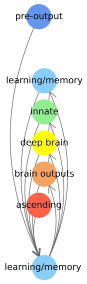


Motif count: 1

**Incoming arcs**:
('ascending', 'learning/memory'): 1.0,
('brain-outputs', 'learning/memory'): 1.0,
('deep-brain', 'learning/memory'): 22.0,
('innate', 'learning/memory'): 22.0,
('learning/memory', 'learning/memory'): 121.0,
('pre-output', 'learning/memory'): 2.0

**Outgoing arcs:**
('learning/memory', 'brain-outputs'): 1.0,
('learning/memory', 'deep-brain'): 5.0,
('learning/memory', 'innate'): 2.0,
('learning/memory', 'learning/memory'): 121.0

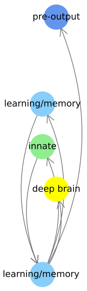


Motif count: 1

**Incoming arcs**:
('innate', 'learning/memory'): 21.0,
('learning/memory', 'learning/memory'): 69.0

**Outgoing arcs**:
('learning/memory', 'deep-brain'): 8.0,
('learning/memory', 'innate'): 1.0,
('learning/memory', 'learning/memory'): 97.0,
('learning/memory', 'pre-output'): 1.0

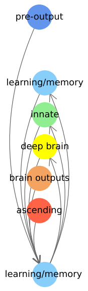


Motif count: 1

**Incoming arcs**:
('ascending', 'learning/memory'): 1.0,
('brain-outputs', 'learning/memory'): 2.0,
('deep-brain', 'learning/memory'): 22.0,
('innate', 'learning/memory'): 31.0,
('learning/memory', 'learning/memory'): 120.0,
('pre-output', 'learning/memory'): 4.0

**Outgoing arcs**:
('learning/memory', 'deep-brain'): 2.0,
('learning/memory', 'innate'): 2.0,
('learning/memory', 'learning/memory'): 108.0

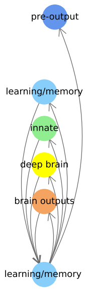


Motif count: 1

**Incoming arcs**:
('brain-outputs', 'learning/memory'): 1.0,
('deep-brain', 'learning/memory'): 4.0,
('innate', 'learning/memory'): 27.0,
('learning/memory', 'learning/memory'): 70.0

**Outgoing arcs**:
('learning/memory', 'brain-outputs'): 5.0,
('learning/memory', 'deep-brain'): 22.0,
('learning/memory', 'innate'): 45.0,
('learning/memory', 'learning/memory'): 4.0,
('learning/memory', 'pre-output'): 9.0

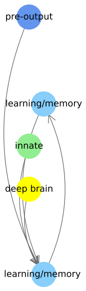


Motif count: 1

**Incoming arcs**:
('deep-brain', 'learning/memory'): 41.0,
('innate', 'learning/memory'): 25.0,
('learning/memory', 'learning/memory'): 105.0,
('pre-output', 'learning/memory'): 28.0

**Outgoing arcs**:
('learning/memory', 'learning/memory'): 114.0

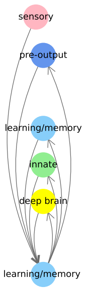


Motif count: 1

**Incoming arcs**:
('deep-brain', 'learning/memory'): 15.0,
('innate', 'learning/memory'): 16.0,
('learning/memory', 'learning/memory'): 50.0,
('pre-output', 'learning/memory'): 4.0,
('sensory', 'learning/memory'): 1.0

**Outgoing arcs**:
('learning/memory', 'deep-brain'): 37.0,
('learning/memory', 'innate'): 20.0,
('learning/memory', 'learning/memory'): 15.0,
('learning/memory', 'pre-output'): 7.0

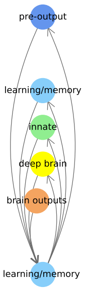


Motif count: 1

**Incoming arcs**:
('brain-outputs', 'learning/memory'): 3.0,
('deep-brain', 'learning/memory'): 41.0,
('innate', 'learning/memory'): 23.0,
('learning/memory', 'learning/memory'): 72.0,
('pre-output', 'learning/memory'): 1.0

**Outgoing arcs**:
('learning/memory', 'deep-brain'): 11.0,
('learning/memory', 'innate'): 2.0,
('learning/memory', 'learning/memory'): 40.0,
('learning/memory', 'pre-output'): 1.0

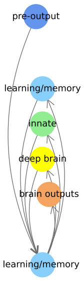


Motif count: 1

**Incoming arcs**:
('deep-brain', 'learning/memory'): 34.0,
('innate', 'learning/memory'): 22.0,
('learning/memory', 'learning/memory'): 72.0,
('pre-output', 'learning/memory'): 3.0

**Outgoing arcs**:
('learning/memory', 'brain-outputs'): 1.0,
('learning/memory', 'deep-brain'): 11.0,
('learning/memory', 'innate'): 6.0,
('learning/memory', 'learning/memory'): 41.0

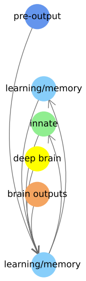


Motif count: 1

**Incoming arcs**:
('brain-outputs', 'learning/memory'): 2.0,
('deep-brain', 'learning/memory'): 26.0,
('innate', 'learning/memory'): 23.0,
('learning/memory', 'learning/memory'): 120.0,
('pre-output', 'learning/memory'): 2.0

**Outgoing arcs**:
('learning/memory', 'innate'): 1.0,
('learning/memory', 'learning/memory'): 108.0

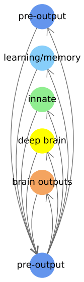


Motif count: 8

**Incoming arcs**:
('brain-outputs', 'pre-output'): 3.0,
('deep-brain', 'pre-output'): 23.62,
('innate', 'pre-output'): 16.75,
('learning/memory', 'pre-output'): 7.75,
('pre-output', 'pre-output'): 26.75

**Outgoing arcs**:
('pre-output', 'brain-outputs'): 8.38,
('pre-output', 'deep-brain'): 26.5,
('pre-output', 'innate'): 7.88,
('pre-output', 'learning/memory'): 5.25,
('pre-output', 'pre-output'): 30.88

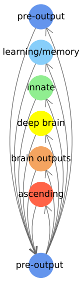


Motif count: 2

**Incoming arcs**:
('ascending', 'pre-output'): 1.0,
('brain-outputs', 'pre-output'): 9.0,
('deep-brain', 'pre-output'): 9.5,
('innate', 'pre-output'): 15.5,
('learning/memory', 'pre-output'): 2.0,
('pre-output', 'pre-output'): 15.0

**Outgoing arcs**:
('pre-output', 'ascending'): 1.0,
('pre-output', 'brain-outputs'): 7.5,
('pre-output', 'deep-brain'): 27.0,
('pre-output', 'innate'): 18.0,
('pre-output', 'learning/memory'): 5.0,
('pre-output', 'pre-output'): 19.0

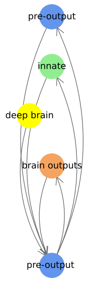


Motif count: 1

**Incoming arcs**:
('brain-outputs', 'pre-output'): 5.0,
('deep-brain', 'pre-output'): 15.0,
('innate', 'pre-output'): 7.0,
('pre-output', 'pre-output'): 57.0

**Outgoing arcs**:
('pre-output', 'brain-outputs'): 12.0,
('pre-output', 'innate'): 12.0,
('pre-output', 'pre-output'): 41.0

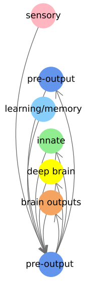


Motif count: 1

**Incoming arcs**:
('brain-outputs', 'pre-output'): 5.0,
('deep-brain', 'pre-output'): 27.0,
('innate', 'pre-output'): 18.0,
('learning/memory', 'pre-output'): 2.0,
('pre-output', 'pre-output'): 32.0,
('sensory', 'pre-output'): 1.0

**Outgoing arcs**:
('pre-output', 'brain-outputs'): 11.0,
('pre-output', 'deep-brain'): 17.0,
('pre-output', 'innate'): 4.0,
('pre-output', 'pre-output'): 29.0

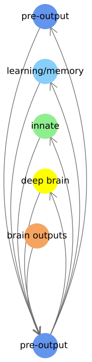


Motif count: 1

**Incoming arcs**:
('brain-outputs', 'pre-output'): 1.0,
('deep-brain', 'pre-output'): 31.0,
('innate', 'pre-output'): 22.0,
('learning/memory', 'pre-output'): 19.0,
('pre-output', 'pre-output'): 6.0

**Outgoing arcs**:
('pre-output', 'deep-brain'): 29.0,
('pre-output', 'innate'): 18.0,
('pre-output', 'learning/memory'): 8.0,
('pre-output', 'pre-output'): 3.0

## 2.2 Adult

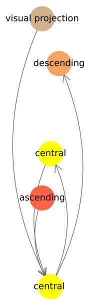


Motif count: 202

**Incoming arcs**:
('ascending', 'central'): 4.37,
('central', 'central'): 74.87,
('visual projection', 'central'): 11.35

**Outgoing arcs**:
('central', 'central'): 52.72,
('central', 'descending'): 4.54

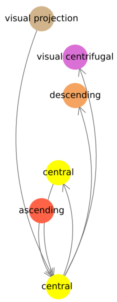


Motif count: 188

**Incoming arcs**:
('ascending', 'central'): 4.43,
('central', 'central'): 71.11,
('visual projection', 'central'): 12.37

**Outgoing arcs**:
('central', 'central'): 50.05,
('central', 'descending'): 6.17,
('central', 'visual centrifugal'): 2.41

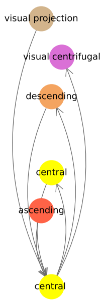


Motif count: 158

**Incoming arcs**:
('ascending', 'central'): 7.94,
('central', 'central'): 88.28,
('descending', 'central'): 5.08,
('visual projection', 'central'): 8.75

**Outgoing arcs**:
('central', 'central'): 51.85,
('central', 'descending'): 12.23,
('central', 'visual centrifugal'): 2.67

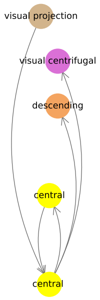


Motif count: 125

**Incoming arcs**:
('central', 'central'): 56.48,
('visual projection', 'central'): 19.43

**Outgoing arcs**:
('central', 'central'): 49.65,
('central', 'descending'): 4.42,
('central', 'visual centrifugal'): 2.26

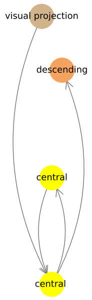


Motif count: 122

**Incoming arcs**:
('central', 'central'): 73.05,
('visual projection', 'central'): 13.83

**Outgoing arcs**:
('central', 'central'): 56.52,
('central', 'descending'): 4.37

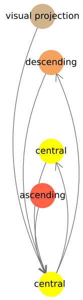


Motif count: 117

**Incoming arcs**:
('ascending', 'central'): 8.38,
('central', 'central'): 102.41,
('descending', 'central'): 3.67,
('visual projection', 'central'): 10.58

**Outgoing arcs**:
('central', 'central'): 56.11,
('central', 'descending'): 10.71

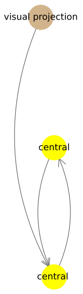


Motif count: 106

**Incoming arcs**:
('central', 'central'): 59.54,
('visual projection', 'central'): 18.73

**Outgoing arcs**:
('central', 'central'): 53.86

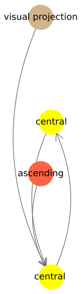


Motif count: 89

**Incoming arcs**:
('ascending', 'central'): 2.93,
('central', 'central'): 67.65,
('visual projection', 'central'): 19.48

**Outgoing arcs**:
('central', 'central'): 57.74

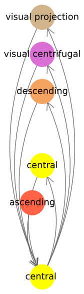


Motif count: 81

**Incoming arcs**:
('ascending', 'central'): 6.41,
('central', 'central'): 135.68,
('descending', 'central'): 4.46,
('visual centrifugal', 'central'): 3.36,
('visual projection', 'central'): 38.85

**Outgoing arcs**:
('central', 'central'): 90.2,
('central', 'descending'): 16.98,
('central', 'visual centrifugal'): 4.8,
('central', 'visual projection'): 10.0

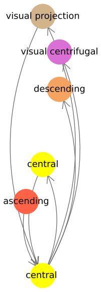


Motif count: 80

**Incoming arcs**:
('ascending', 'central'): 2.98,
('central', 'central'): 81.16,
('visual projection', 'central'): 26.79

**Outgoing arcs**:
('central', 'central'): 82.19,
('central', 'descending'): 7.1,
('central', 'visual centrifugal'): 3.27,
('central', 'visual projection'): 10.19

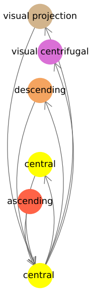


Motif count: 70

**Incoming arcs**:
('ascending', 'central'): 6.49,
('central', 'central'): 79.6,
('descending', 'central'): 3.97,
('visual projection', 'central'): 25.29

**Outgoing arcs**:
('central', 'central'): 96.43,
('central', 'descending'): 12.07,
('central', 'visual centrifugal'): 5.37,
('central', 'visual projection'): 4.9

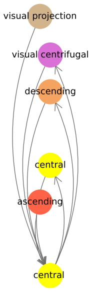


Motif count: 67

**Incoming arcs**:
('ascending', 'central'): 6.1,
('central', 'central'): 107.72,
('descending', 'central'): 3.93,
('visual centrifugal', 'central'): 2.43,
('visual projection', 'central'): 19.55

**Outgoing arcs**:
('central', 'central'): 59.21,
('central', 'descending'): 15.19,
('central', 'visual centrifugal'): 3.36

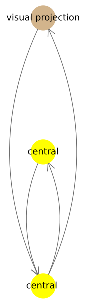


Motif count: 61

**Incoming arcs**:
('central', 'central'): 77.23,
('visual projection', 'central'): 49.18

**Outgoing arcs**:
('central', 'central'): 94.49,
('central', 'visual projection'): 24.64

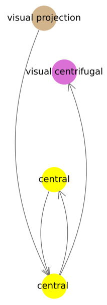


Motif count: 58

**Incoming arcs**:
('central', 'central'): 36.57,
('visual projection', 'central'): 31.67

**Outgoing arcs**:
('central', 'central'): 46.22,
('central', 'visual centrifugal'): 2.03

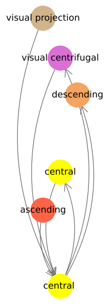


Motif count: 58

**Incoming arcs**:
('ascending', 'central'): 3.52,
('central', 'central'): 73.52,
('visual centrifugal', 'central'): 2.24,
('visual projection', 'central'): 23.79

**Outgoing arcs**:
('central', 'central'): 53.81,
('central', 'descending'): 8.45,
('central', 'visual centrifugal'): 3.34

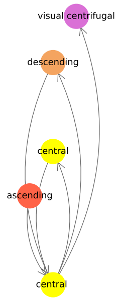


Motif count: 51

**Incoming arcs**:
('ascending', 'central'): 10.25,
('central', 'central'): 59.12,
('descending', 'central'): 6.86

**Outgoing arcs**:
('central', 'central'): 59.02,
('central', 'descending'): 13.67,
('central', 'visual centrifugal'): 4.14

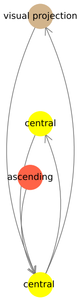


Motif count: 50

**Incoming arcs**:
('ascending', 'central'): 2.04,
('central', 'central'): 42.36,
('visual projection', 'central'): 56.12

**Outgoing arcs**:
('central', 'central'): 59.3,
('central', 'visual projection'): 15.82

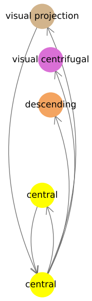


Motif count: 48

**Incoming arcs**:
('central', 'central'): 52.23,
('visual projection', 'central'): 31.08

**Outgoing arcs**:
('central', 'central'): 60.4,
('central', 'descending'): 3.48,
('central', 'visual centrifugal'): 2.29,
('central', 'visual projection'): 2.79

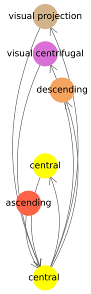


Motif count: 45

**Incoming arcs**:
('ascending', 'central'): 3.64,
('central', 'central'): 72.87,
('visual centrifugal', 'central'): 2.82,
('visual projection', 'central'): 55.36

**Outgoing arcs**:
('central', 'central'): 82.53,
('central', 'descending'): 7.51,
('central', 'visual centrifugal'): 3.8,
('central', 'visual projection'): 20.4

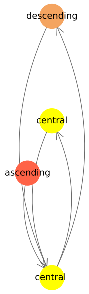


Motif count: 45

**Incoming arcs**:
('ascending', 'central'): 8.38,
('central', 'central'): 89.18,
('descending', 'central'): 4.69

**Outgoing arcs**:
('central', 'central'): 63.6,
('central', 'descending'): 9.78

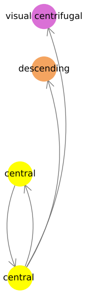


Motif count: 44

**Incoming arcs**:
('central', 'central'): 55.11

**Outgoing arcs**:
('central', 'central'): 43.05,
('central', 'descending'): 4.77,
('central', 'visual centrifugal'): 2.52

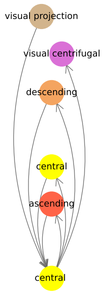


Motif count: 43

**Incoming arcs**:
('ascending', 'central'): 11.16,
('central', 'central'): 80.63,
('descending', 'central'): 4.88,
('visual projection', 'central'): 8.6

**Outgoing arcs**:
('central', 'ascending'): 2.6,
('central', 'central'): 92.56,
('central', 'descending'): 14.72,
('central', 'visual centrifugal'): 2.35

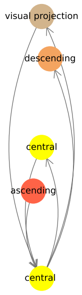


Motif count: 43

**Incoming arcs**:
('ascending', 'central'): 3.02,
('central', 'central'): 64.56,
('visual projection', 'central'): 34.93

**Outgoing arcs**:
('central', 'central'): 71.14,
('central', 'descending'): 4.02,
('central', 'visual projection'): 12.4

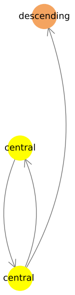


Motif count: 38

**Incoming arcs**:
('central', 'central'): 82.37

**Outgoing arcs**:
('central', 'central'): 61.08,
('central', 'descending'): 4.34

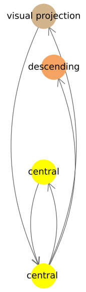


Motif count: 37

**Incoming arcs**:
('central', 'central'): 61.54,
('visual projection', 'central'): 32.0

**Outgoing arcs**:
('central', 'central'): 64.84,
('central', 'descending'): 4.32,
('central', 'visual projection'): 7.35

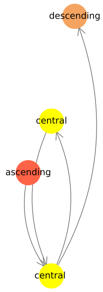


Motif count: 36

**Incoming arcs**:
('ascending', 'central'): 2.94,
('central', 'central'): 90.89

**Outgoing arcs**:
('central', 'central'): 69.31,
('central', 'descending'): 6.56

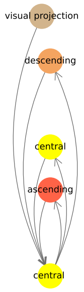


Motif count: 36

**Incoming arcs**:
('ascending', 'central'): 12.81,
('central', 'central'): 104.19,
('descending', 'central'): 6.67,
('visual projection', 'central'): 4.72

**Outgoing arcs**:
('central', 'ascending'): 3.58,
('central', 'central'): 105.17,
('central', 'descending'): 14.78

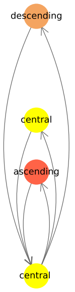


Motif count: 33

**Incoming arcs**:
('ascending', 'central'): 14.39,
('central', 'central'): 48.82,
('descending', 'central'): 8.09

**Outgoing arcs**:
('central', 'ascending'): 2.76,
('central', 'central'): 48.03,
('central', 'descending'): 17.48

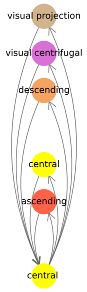


Motif count: 32

**Incoming arcs**:
('ascending', 'central'): 11.09,
('central', 'central'): 172.62,
('descending', 'central'): 3.97,
('visual centrifugal', 'central'): 2.25,
('visual projection', 'central'): 69.78

**Outgoing arcs**:
('central', 'ascending'): 3.81,
('central', 'central'): 196.88,
('central', 'descending'): 12.56,
('central', 'visual centrifugal'): 4.53,
('central', 'visual projection'): 34.34

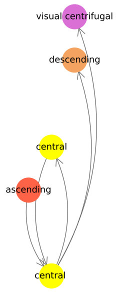


Motif count: 31

**Incoming arcs**:
('ascending', 'central'): 3.68,
('central', 'central'): 61.39

**Outgoing arcs**:
('central', 'central'): 53.32,
('central', 'descending'): 7.06,
('central', 'visual centrifugal'): 2.35

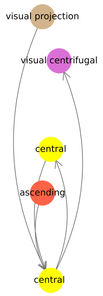


Motif count: 31

**Incoming arcs**:
('ascending', 'central'): 3.61,
('central', 'central'): 72.74,
('visual projection', 'central'): 35.23

**Outgoing arcs**:
('central', 'central'): 72.81,
('central', 'visual centrifugal'): 2.52

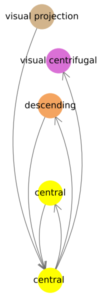


Motif count: 30

**Incoming arcs**:
('central', 'central'): 84.3,
('descending', 'central'): 1.83,
('visual projection', 'central'): 28.67

**Outgoing arcs**:
('central', 'central'): 60.8,
('central', 'descending'): 7.77,
('central', 'visual centrifugal'): 2.33

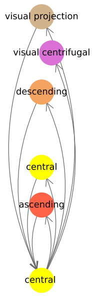


Motif count: 29

**Incoming arcs**:
('ascending', 'central'): 12.28,
('central', 'central'): 142.93,
('descending', 'central'): 4.55,
('visual projection', 'central'): 37.97

**Outgoing arcs**:
('central', 'ascending'): 3.38,
('central', 'central'): 174.66,
('central', 'descending'): 12.1,
('central', 'visual centrifugal'): 3.28,
('central', 'visual projection'): 13.1

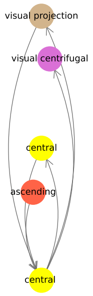


Motif count: 25

**Incoming arcs**:
('ascending', 'central'): 2.56,
('central', 'central'): 33.2,
('visual projection', 'central'): 89.04

**Outgoing arcs**:
('central', 'central'): 50.8,
('central', 'visual centrifugal'): 1.28,
('central', 'visual projection'): 23.72

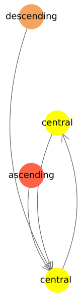


Motif count: 25

**Incoming arcs**:
('ascending', 'central'): 2.8,
('central', 'central'): 109.08,
('descending', 'central'): 2.36

**Outgoing arcs**:
('central', 'central'): 71.96

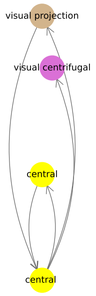


Motif count: 24

**Incoming arcs**:
('central', 'central'): 37.17,
('visual projection', 'central'): 44.29

**Outgoing arcs**:
('central', 'central'): 52.08,
('central', 'visual centrifugal'): 1.62,
('central', 'visual projection'): 15.54

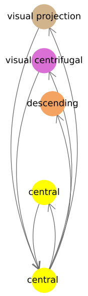


Motif count: 23

**Incoming arcs**:
('central', 'central'): 64.65,
('visual centrifugal', 'central'): 1.83,
('visual projection', 'central'): 44.17

**Outgoing arcs**:
('central', 'central'): 64.26,
('central', 'descending'): 4.48,
('central', 'visual centrifugal'): 2.91,
('central', 'visual projection'): 11.7

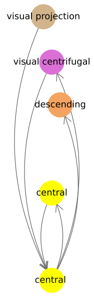


Motif count: 23

**Incoming arcs**:
('central', 'central'): 47.3,
('visual centrifugal', 'central'): 1.74,
('visual projection', 'central'): 40.91

**Outgoing arcs**:
('central', 'central'): 40.91,
('central', 'descending'): 5.96,
('central', 'visual centrifugal'): 2.91

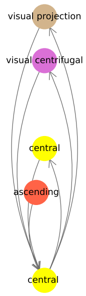


Motif count: 22

**Incoming arcs**:
('ascending', 'central'): 2.68,
('central', 'central'): 59.73,
('visual centrifugal', 'central'): 1.41,
('visual projection', 'central'): 49.18

**Outgoing arcs**:
('central', 'central'): 68.95,
('central', 'visual centrifugal'): 2.23,
('central', 'visual projection'): 11.73

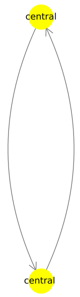


Motif count: 21

**Incoming arcs**:
('central', 'central'): 114.67

**Outgoing arcs**:
('central', 'central'): 100.14

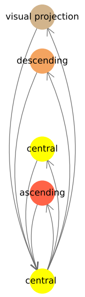


Motif count: 21

**Incoming arcs**:
('ascending', 'central'): 10.76,
('central', 'central'): 114.95,
('descending', 'central'): 3.62,
('visual projection', 'central'): 7.19

**Outgoing arcs**:
('central', 'ascending'): 3.95,
('central', 'central'): 121.48,
('central', 'descending'): 7.62,
('central', 'visual projection'): 3.48

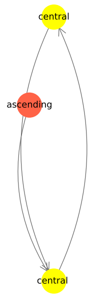


Motif count: 20

**Incoming arcs**:
('ascending', 'central'): 3.15,
('central', 'central'): 70.65

**Outgoing arcs**:
('central', 'central'): 68.25

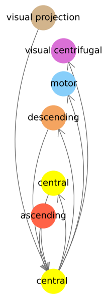


Motif count: 20

**Incoming arcs**:
('ascending', 'central'): 8.0,
('central', 'central'): 89.45,
('descending', 'central'): 10.8,
('visual projection', 'central'): 14.65

**Outgoing arcs**:
('central', 'central'): 42.2,
('central', 'descending'): 20.35,
('central', 'motor'): 2.0,
('central', 'visual centrifugal'): 5.25

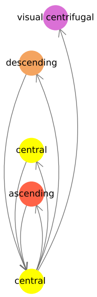


Motif count: 19

**Incoming arcs**:
('ascending', 'central'): 12.53,
('central', 'central'): 77.16,
('descending', 'central'): 10.0

**Outgoing arcs**:
('central', 'ascending'): 2.95,
('central', 'central'): 80.68,
('central', 'descending'): 16.53,
('central', 'visual centrifugal'): 3.79

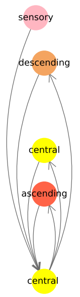


Motif count: 19

**Incoming arcs**:
('ascending', 'central'): 10.53,
('central', 'central'): 52.05,
('descending', 'central'): 4.58,
('sensory', 'central'): 11.68

**Outgoing arcs**:
('central', 'ascending'): 5.95,
('central', 'central'): 77.68,
('central', 'descending'): 15.0

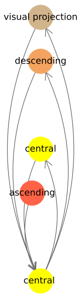


Motif count: 19

**Incoming arcs**:
('ascending', 'central'): 7.42,
('central', 'central'): 80.37,
('descending', 'central'): 2.84,
('visual projection', 'central'): 16.89

**Outgoing arcs**:
('central', 'central'): 61.42,
('central', 'descending'): 8.84,
('central', 'visual projection'): 2.63

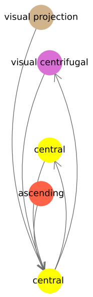


Motif count: 19

**Incoming arcs**:
('ascending', 'central'): 2.79,
('central', 'central'): 59.89,
('visual centrifugal', 'central'): 1.32,
('visual projection', 'central'): 34.84

**Outgoing arcs**:
('central', 'central'): 61.74,
('central', 'visual centrifugal'): 1.95

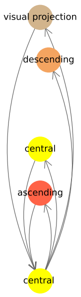


Motif count: 19

**Incoming arcs**:
('ascending', 'central'): 5.16,
('central', 'central'): 114.0,
('visual projection', 'central'): 17.68

**Outgoing arcs**:
('central', 'ascending'): 1.79,
('central', 'central'): 127.21,
('central', 'descending'): 3.05,
('central', 'visual projection'): 6.89

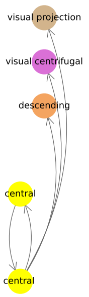


Motif count: 18

**Incoming arcs**:
('central', 'central'): 56.94

**Outgoing arcs**:
('central', 'central'): 47.17,
('central', 'descending'): 6.56,
('central', 'visual centrifugal'): 1.78,
('central', 'visual projection'): 2.5

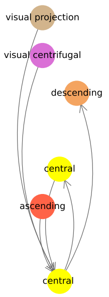


Motif count: 18

**Incoming arcs**:
('ascending', 'central'): 4.39,
('central', 'central'): 51.0,
('visual centrifugal', 'central'): 2.0,
('visual projection', 'central'): 25.17

**Outgoing arcs**:
('central', 'central'): 40.06,
('central', 'descending'): 9.0

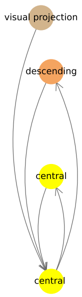


Motif count: 18

**Incoming arcs**:
('central', 'central'): 115.83,
('descending', 'central'): 1.5,
('visual projection', 'central'): 14.28

**Outgoing arcs**:
('central', 'central'): 81.28,
('central', 'descending'): 5.61

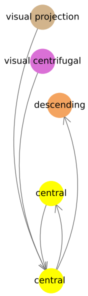


Motif count: 17

**Incoming arcs**:
('central', 'central'): 48.47,
('visual centrifugal', 'central'): 1.24,
('visual projection', 'central'): 22.47

**Outgoing arcs**:
('central', 'central'): 36.53,
('central', 'descending'): 5.18

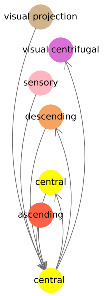


Motif count: 16

**Incoming arcs**:
('ascending', 'central'): 10.0,
('central', 'central'): 64.12,
('descending', 'central'): 5.56,
('sensory', 'central'): 3.06,
('visual projection', 'central'): 9.31

**Outgoing arcs**:
('central', 'central'): 58.25,
('central', 'descending'): 9.94,
('central', 'visual centrifugal'): 3.31

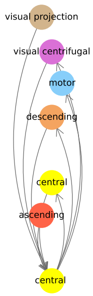


Motif count: 16

**Incoming arcs**:
('ascending', 'central'): 4.69,
('central', 'central'): 74.06,
('descending', 'central'): 7.62,
('visual centrifugal', 'central'): 1.12,
('visual projection', 'central'): 9.25

**Outgoing arcs**:
('central', 'central'): 30.75,
('central', 'descending'): 19.12,
('central', 'motor'): 1.94,
('central', 'visual centrifugal'): 3.75

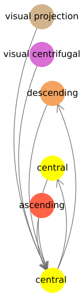


Motif count: 15

**Incoming arcs**:
('ascending', 'central'): 6.2,
('central', 'central'): 90.8,
('descending', 'central'): 3.4,
('visual centrifugal', 'central'): 1.47,
('visual projection', 'central'): 5.07

**Outgoing arcs**:
('central', 'central'): 54.4,
('central', 'descending'): 13.53

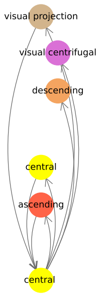


Motif count: 15

**Incoming arcs**:
('ascending', 'central'): 6.73,
('central', 'central'): 89.07,
('visual projection', 'central'): 27.47

**Outgoing arcs**:
('central', 'ascending'): 3.6,
('central', 'central'): 132.47,
('central', 'descending'): 7.53,
('central', 'visual centrifugal'): 2.33,
('central', 'visual projection'): 7.2

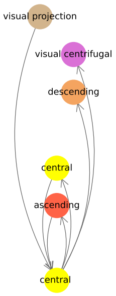


Motif count: 14

**Incoming arcs**:
('ascending', 'central'): 6.93,
('central', 'central'): 142.93,
('visual projection', 'central'): 13.5

**Outgoing arcs**:
('central', 'ascending'): 3.29,
('central', 'central'): 94.07,
('central', 'descending'): 8.14,
('central', 'visual centrifugal'): 1.71

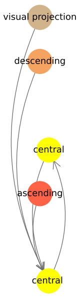


Motif count: 14

**Incoming arcs**:
('ascending', 'central'): 1.57,
('central', 'central'): 127.36,
('descending', 'central'): 1.5,
('visual projection', 'central'): 7.93

**Outgoing arcs**:
('central', 'central'): 76.29

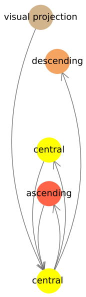


Motif count: 14

**Incoming arcs**:
('ascending', 'central'): 7.79,
('central', 'central'): 56.36,
('visual projection', 'central'): 5.0

**Outgoing arcs**:
('central', 'ascending'): 2.14,
('central', 'central'): 76.57,
('central', 'descending'): 3.93

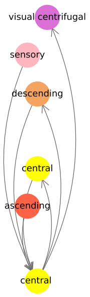


Motif count: 13

**Incoming arcs**:
('ascending', 'central'): 10.69,
('central', 'central'): 70.46,
('descending', 'central'): 9.69,
('sensory', 'central'): 6.23

**Outgoing arcs**:
('central', 'central'): 74.38,
('central', 'descending'): 13.77,
('central', 'visual centrifugal'): 3.08

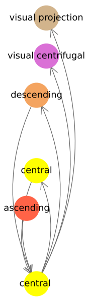


Motif count: 12

**Incoming arcs**:
('ascending', 'central'): 15.08,
('central', 'central'): 50.17,
('descending', 'central'): 12.08

**Outgoing arcs**:
('central', 'central'): 148.5,
('central', 'descending'): 19.08,
('central', 'visual centrifugal'): 7.5,
('central', 'visual projection'): 1.5

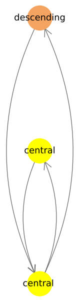


Motif count: 12

**Incoming arcs**:
('central', 'central'): 68.83,
('descending', 'central'): 1.5

**Outgoing arcs**:
('central', 'central'): 60.92,
('central', 'descending'): 7.5

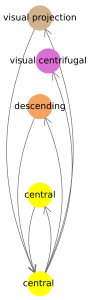


Motif count: 12

**Incoming arcs**:
('central', 'central'): 91.58,
('descending', 'central'): 1.42,
('visual projection', 'central'): 25.67

**Outgoing arcs**:
('central', 'central'): 139.17,
('central', 'descending'): 7.42,
('central', 'visual centrifugal'): 2.58,
('central', 'visual projection'): 4.25

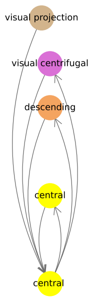


Motif count: 12

**Incoming arcs**:
('central', 'central'): 110.75,
('descending', 'central'): 2.25,
('visual centrifugal', 'central'): 1.42,
('visual projection', 'central'): 15.08

**Outgoing arcs**:
('central', 'central'): 66.5,
('central', 'descending'): 8.42,
('central', 'visual centrifugal'): 3.58

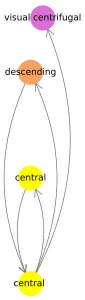


Motif count: 12

**Incoming arcs**:
('central', 'central'): 53.75,
('descending', 'central'): 1.58

**Outgoing arcs**:
('central', 'central'): 46.0,
('central', 'descending'): 5.75,
('central', 'visual centrifugal'): 2.33

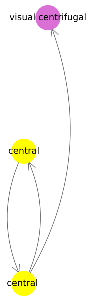


Motif count: 11

**Incoming arcs**:
('central', 'central'): 68.09

**Outgoing arcs**:
('central', 'central'): 72.73,
('central', 'visual centrifugal'): 1.45

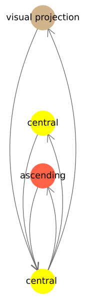


Motif count: 11

**Incoming arcs**:
('ascending', 'central'): 4.18,
('central', 'central'): 38.55,
('visual projection', 'central'): 35.09

**Outgoing arcs**:
('central', 'ascending'): 2.27,
('central', 'central'): 62.64,
('central', 'visual projection'): 22.36

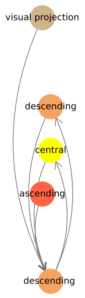


Motif count: 21

**Incoming arcs**:
('ascending', 'descending'): 18.0,
('central', 'descending'): 123.62,
('descending', 'descending'): 19.76,
('visual projection', 'descending'): 5.81

**Outgoing arcs**:
('descending', 'central'): 21.19,
('descending', 'descending'): 17.67

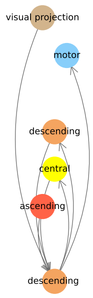


Motif count: 21

**Incoming arcs**:
('ascending', 'descending'): 7.81,
('central', 'descending'): 82.95,
('descending', 'descending'): 11.67,
('visual projection', 'descending'): 7.9

**Outgoing arcs**:
('descending', 'central'): 17.05,
('descending', 'descending'): 13.76,
('descending', 'motor'): 3.19

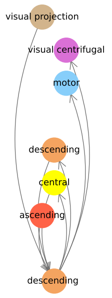


Motif count: 20

**Incoming arcs**:
('ascending', 'descending'): 13.8,
('central', 'descending'): 101.15,
('descending', 'descending'): 16.95,
('visual projection', 'descending'): 9.35

**Outgoing arcs**:
('descending', 'central'): 27.1,
('descending', 'descending'): 24.0,
('descending', 'motor'): 4.8,
('descending', 'visual centrifugal'): 1.9

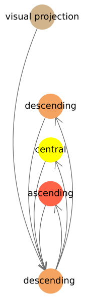


Motif count: 20

**Incoming arcs**:
('ascending', 'descending'): 15.15,
('central', 'descending'): 86.2,
('descending', 'descending'): 12.8,
('visual projection', 'descending'): 4.55

**Outgoing arcs**:
('descending', 'ascending'): 2.65,
('descending', 'central'): 31.1,
('descending', 'descending'): 17.1

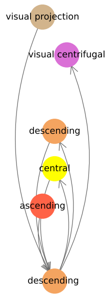


Motif count: 17

**Incoming arcs**:
('ascending', 'descending'): 9.47,
('central', 'descending'): 157.41,
('descending', 'descending'): 5.82,
('visual projection', 'descending'): 10.59

**Outgoing arcs**:
('descending', 'central'): 40.94,
('descending', 'descending'): 16.53,
('descending', 'visual centrifugal'): 3.88

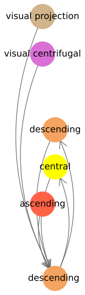


Motif count: 14

**Incoming arcs**:
('ascending', 'descending'): 12.79,
('central', 'descending'): 183.86,
('descending', 'descending'): 11.29,
('visual centrifugal', 'descending'): 1.86,
('visual projection', 'descending'): 45.0

**Outgoing arcs**:
('descending', 'central'): 17.43,
('descending', 'descending'): 12.21

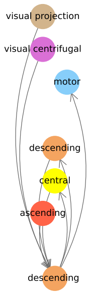


Motif count: 14

**Incoming arcs**:
('ascending', 'descending'): 8.5,
('central', 'descending'): 135.29,
('descending', 'descending'): 10.0,
('visual centrifugal', 'descending'): 5.36,
('visual projection', 'descending'): 50.57

**Outgoing arcs**:
('descending', 'central'): 17.0,
('descending', 'descending'): 11.07,
('descending', 'motor'): 1.79

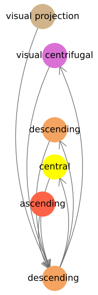


Motif count: 13

**Incoming arcs**:
('ascending', 'descending'): 5.85,
('central', 'descending'): 135.31,
('descending', 'descending'): 4.69,
('visual centrifugal', 'descending'): 2.85,
('visual projection', 'descending'): 15.77

**Outgoing arcs**:
('descending', 'central'): 48.0,
('descending', 'descending'): 16.0,
('descending', 'visual centrifugal'): 3.77

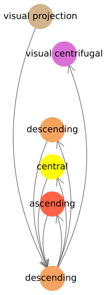


Motif count: 12

**Incoming arcs**:
('ascending', 'descending'): 12.25,
('central', 'descending'): 123.58,
('descending', 'descending'): 3.0,
('visual projection', 'descending'): 12.42

**Outgoing arcs**:
('descending', 'ascending'): 2.67,
('descending', 'central'): 40.83,
('descending', 'descending'): 18.75,
('descending', 'visual centrifugal'): 3.75

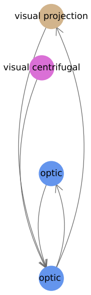


Motif count: 122

**Incoming arcs**:
('optic', 'optic'): 154.88,
('visual centrifugal', 'optic'): 7.46,
('visual projection', 'optic'): 19.91

**Outgoing arcs**:
('optic', 'optic'): 66.73,
('optic', 'visual projection'): 43.62

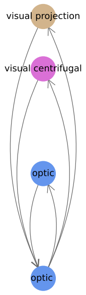


Motif count: 79

**Incoming arcs**:
('optic', 'optic'): 541.75,
('visual centrifugal', 'optic'): 8.78,
('visual projection', 'optic'): 20.77

**Outgoing arcs**:
('optic', 'optic'): 254.52,
('optic', 'visual centrifugal'): 2.51,
('optic', 'visual projection'): 91.68

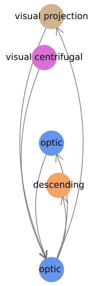


Motif count: 17

**Incoming arcs**:
('optic', 'optic'): 194.35,
('visual centrifugal', 'optic'): 7.0,
('visual projection', 'optic'): 34.41

**Outgoing arcs**:
('optic', 'descending'): 1.0,
('optic', 'optic'): 95.94,
('optic', 'visual projection'): 77.59

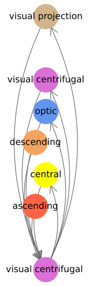


Motif count: 23

**Incoming arcs**:
('ascending', 'visual centrifugal'): 5.57,
('central', 'visual centrifugal'): 78.04,
('descending', 'visual centrifugal'): 4.26,
('optic', 'visual centrifugal'): 17.83,
('visual centrifugal', 'visual centrifugal'): 9.74,
('visual projection', 'visual centrifugal'): 94.26

**Outgoing arcs**:
('visual centrifugal', 'central'): 7.57,
('visual centrifugal', 'optic'): 399.17,
('visual centrifugal', 'visual centrifugal'): 5.96,
('visual centrifugal', 'visual projection'): 95.57

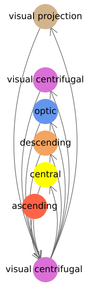


Motif count: 19

**Incoming arcs**:
('ascending', 'visual centrifugal'): 6.68,
('central', 'visual centrifugal'): 137.89,
('descending', 'visual centrifugal'): 6.21,
('optic', 'visual centrifugal'): 8.58,
('visual centrifugal', 'visual centrifugal'): 6.42,
('visual projection', 'visual centrifugal'): 50.47

**Outgoing arcs**:
('visual centrifugal', 'central'): 57.37,
('visual centrifugal', 'descending'): 6.21,
('visual centrifugal', 'optic'): 103.79,
('visual centrifugal', 'visual centrifugal'): 9.95,
('visual centrifugal', 'visual projection'): 119.74

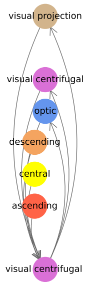


Motif count: 15

**Incoming arcs**:
('ascending', 'visual centrifugal'): 7.47,
('central', 'visual centrifugal'): 58.67,
('descending', 'visual centrifugal'): 7.2,
('optic', 'visual centrifugal'): 198.47,
('visual centrifugal', 'visual centrifugal'): 4.67,
('visual projection', 'visual centrifugal'): 15.07

**Outgoing arcs**:
('visual centrifugal', 'optic'): 743.0,
('visual centrifugal', 'visual centrifugal'): 6.0,
('visual centrifugal', 'visual projection'): 71.33

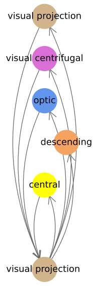


Motif count: 197

**Incoming arcs**:
('central', 'visual projection'): 5.78,
('optic', 'visual projection'): 96.06,
('visual centrifugal', 'visual projection'): 7.54,
('visual projection', 'visual projection'): 13.07

**Outgoing arcs**:
('visual projection', 'central'): 39.4,
('visual projection', 'descending'): 3.5,
('visual projection', 'optic'): 9.44,
('visual projection', 'visual centrifugal'): 3.4,
('visual projection', 'visual projection'): 11.26

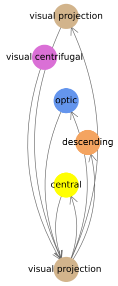


Motif count: 103

**Incoming arcs**:
('central', 'visual projection'): 4.8,
('optic', 'visual projection'): 99.4,
('visual centrifugal', 'visual projection'): 4.0,
('visual projection', 'visual projection'): 3.42

**Outgoing arcs**:
('visual projection', 'central'): 13.7,
('visual projection', 'descending'): 2.91,
('visual projection', 'optic'): 5.12,
('visual projection', 'visual projection'): 7.02

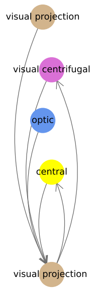


Motif count: 102

**Incoming arcs**:
('central', 'visual projection'): 2.75,
('optic', 'visual projection'): 63.15,
('visual centrifugal', 'visual projection'): 3.5,
('visual projection', 'visual projection'): 4.8

**Outgoing arcs**:
('visual projection', 'central'): 19.18,
('visual projection', 'visual centrifugal'): 2.0

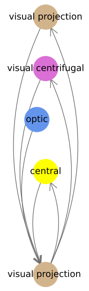


Motif count: 85

**Incoming arcs**:
('central', 'visual projection'): 4.34,
('optic', 'visual projection'): 57.38,
('visual centrifugal', 'visual projection'): 3.41,
('visual projection', 'visual projection'): 8.95

**Outgoing arcs**:
('visual projection', 'central'): 25.95,
('visual projection', 'visual centrifugal'): 2.01,
('visual projection', 'visual projection'): 1.74

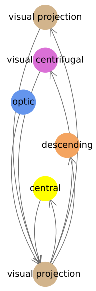


Motif count: 80

**Incoming arcs**:
('central', 'visual projection'): 9.11,
('optic', 'visual projection'): 67.17,
('visual centrifugal', 'visual projection'): 6.55,
('visual projection', 'visual projection'): 32.02

**Outgoing arcs**:
('visual projection', 'central'): 43.81,
('visual projection', 'descending'): 5.76,
('visual projection', 'visual centrifugal'): 2.92,
('visual projection', 'visual projection'): 5.54

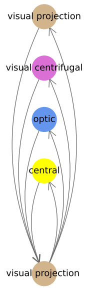


Motif count: 69

**Incoming arcs**:
('central', 'visual projection'): 6.64,
('optic', 'visual projection'): 101.07,
('visual centrifugal', 'visual projection'): 4.8,
('visual projection', 'visual projection'): 15.1

**Outgoing arcs**:
('visual projection', 'central'): 39.59,
('visual projection', 'optic'): 15.38,
('visual projection', 'visual centrifugal'): 2.48,
('visual projection', 'visual projection'): 17.09

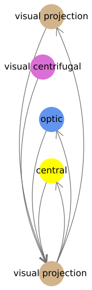


Motif count: 43

**Incoming arcs**:
('central', 'visual projection'): 6.53,
('optic', 'visual projection'): 81.49,
('visual centrifugal', 'visual projection'): 5.79,
('visual projection', 'visual projection'): 14.33

**Outgoing arcs**:
('visual projection', 'central'): 24.37,
('visual projection', 'optic'): 21.56,
('visual projection', 'visual projection'): 22.16

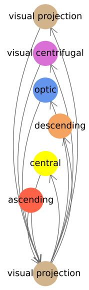


Motif count: 31

**Incoming arcs**:
('ascending', 'visual projection'): 2.1,
('central', 'visual projection'): 15.65,
('optic', 'visual projection'): 135.48,
('visual centrifugal', 'visual projection'): 7.52,
('visual projection', 'visual projection'): 29.84

**Outgoing arcs**:
('visual projection', 'central'): 51.23,
('visual projection', 'descending'): 7.71,
('visual projection', 'optic'): 7.68,
('visual projection', 'visual centrifugal'): 2.48,
('visual projection', 'visual projection'): 8.61

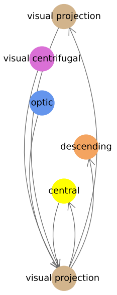


Motif count: 19

**Incoming arcs**:
('central', 'visual projection'): 8.84,
('optic', 'visual projection'): 35.37,
('visual centrifugal', 'visual projection'): 2.47,
('visual projection', 'visual projection'): 12.89

**Outgoing arcs**:
('visual projection', 'central'): 30.0,
('visual projection', 'descending'): 3.68,
('visual projection', 'visual projection'): 4.0

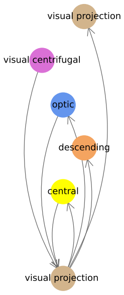


Motif count: 16

**Incoming arcs**:
('central', 'visual projection'): 4.62,
('optic', 'visual projection'): 90.75,
('visual centrifugal', 'visual projection'): 2.94

**Outgoing arcs**:
('visual projection', 'central'): 8.44,
('visual projection', 'descending'): 3.19,
('visual projection', 'optic'): 4.0,
('visual projection', 'visual projection'): 5.69

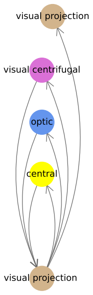


Motif count: 15

**Incoming arcs**:
('central', 'visual projection'): 3.33,
('optic', 'visual projection'): 30.13,
('visual centrifugal', 'visual projection'): 3.6

**Outgoing arcs**:
('visual projection', 'central'): 34.0,
('visual projection', 'optic'): 3.27,
('visual projection', 'visual centrifugal'): 1.87,
('visual projection', 'visual projection'): 4.53

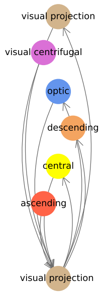


Motif count: 11

**Incoming arcs**:
('ascending', 'visual projection'): 2.09,
('central', 'visual projection'): 12.82,
('optic', 'visual projection'): 43.91,
('visual centrifugal', 'visual projection'): 3.55,
('visual projection', 'visual projection'): 21.73

**Outgoing arcs**:
('visual projection', 'central'): 24.64,
('visual projection', 'descending'): 4.18,
('visual projection', 'optic'): 2.36,
('visual projection', 'visual projection'): 9.91

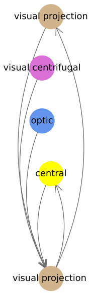


Motif count: 11

**Incoming arcs**:
('central', 'visual projection'): 8.18,
('optic', 'visual projection'): 34.0,
('visual centrifugal', 'visual projection'): 3.45,
('visual projection', 'visual projection'): 19.64

**Outgoing arcs**:
('visual projection', 'central'): 60.45,
('visual projection', 'visual projection'): 5.18
